# Discovery of Novel Pyrrolidine-Based Chalcones as Dual Inhibitors of α-Amylase and α-Glucosidase: Synthesis, Molecular Docking, ADMET Profiles, and Pharmacological Screening

**DOI:** 10.1021/acsomega.4c10095

**Published:** 2025-02-26

**Authors:** Bedriye Seda Kurşun Aktar, Yusuf Sıcak, Abdulkadir Bakırdöven, Gizem Tatar Yılmaz, Özlem Yılmaz, Ayşegül Karaküçük-İyidoğan, Demet Taşdemir, Ebru Sağlam, Emine Elçin Oruç-Emre

**Affiliations:** † Department of Hair Care and Beauty Services, Yeşilyurt Vocational School, Malatya Turgut Özal University, Malatya 44090, Türkiye; ‡ Department of Medicinal and Aromatic Plants, Köyceğiz Vocational School, Mugla Sitki Kocman University, Köyceğiz, Muğla 48000, Türkiye; § Department of Biostatistics and Medical Informatics, Faculty of Medicine, Karadeniz Technical University, Trabzon 61080, Türkiye; ∥ Department of Bioinformatics, Institute of Health Sciences, Karadeniz Technical University, Trabzon 61080, Türkiye; ⊥ Yılmaz Bilişim R&D Consulting Software Engineering and Services Trade Limited Company, Trabzon 61081, Türkiye; # Department of Chemistry, Faculty of Arts and Sciences, Tokat Gaziosmanpaşa University, Tokat 60250, Türkiye; ¶ Department of Chemistry, Faculty of Arts and Sciences, Gaziantep University, Gaziantep 27410, Türkiye; ∇ Department of Medical Biochemistry, Faculty of Medicine, Gaziantep University, Gaziantep 27410, Türkiye; ○ Respiratory Diseases and Respiratory Surgery Research and Practice Center, Gaziantep University, Gaziantep 27410, Türkiye

## Abstract

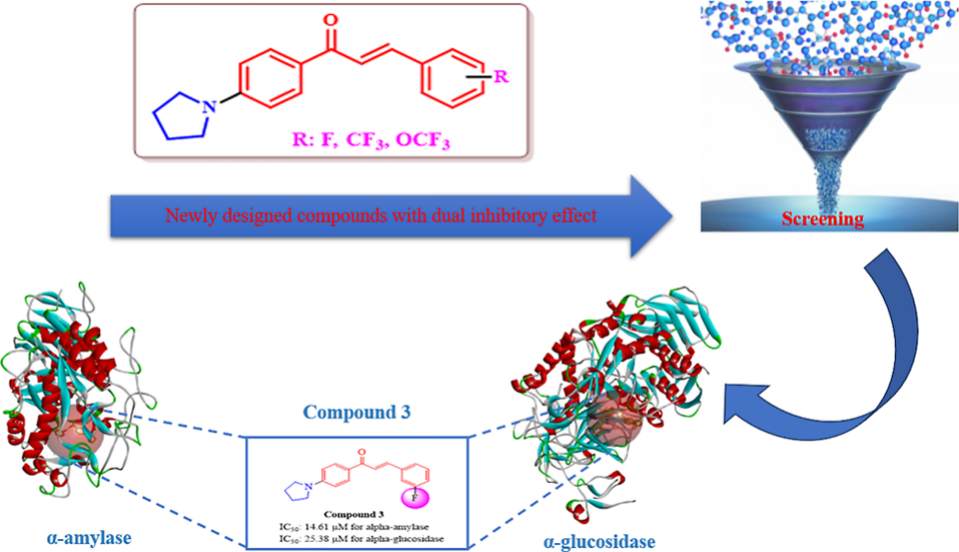

A series of chalcones containing a pyrrolidine moiety were synthesized to examine their *in vitro* α-amylase and α-glucosidase inhibitory activities, for the treatment of *Diabetes mellitus*, which is one of the most dangerous and rapidly increasing disorders of today. Compound **3** exhibited an excellent dual inhibitory effect with an IC_50_ value of 14.61 ± 0.12 μM against α-amylase, and with an IC_50_: 25.38 ± 2.09 μM against α-glucosidase. The *in vitro* cytotoxic effects of all compounds against nonsmall lung cancer (A549) and bronchial epithelial normal (BEAS-2B) cell lines were also evaluated. Compound **5** (IC_50_: 82.20 μM) and compound **8** (IC_50_: 59.96 μM) showed better cytotoxic activity than cisplatin against A549 (IC_50_: 84.39 μM) cells. Furthermore, these compounds had no harmful effect on healthy BEAS-2B cells at the determined IC_50_ values. Moreover, the molecular docking and molecular dynamics simulation analysis revealed that all synthesized compounds exhibited stronger binding affinities toward α-glucosidase and α-amylase compared to the positive control acarbose.

## 1 Introduction

*Diabetes mellitus* (DM) is a systemic and metabolic disorder that progresses with increased blood glucose levels due to inadequate insulin secretion or insulin resistance and can cause momentous damage to vital organs such as the heart, eyes, blood vessels, nerves, and kidneys, over time.^1,2^ The pathophysiology of Type 2 *Diabetes mellitus* (T2DM), the most widespread kind of DM and typically manifesting in adults, is defined by decreased peripheral insulin sensitivity, lack of effective glucose uptake in target tissues such as skeletal muscle, adipose tissue and liver, dysregulation of hepatic glucose production, and decreased β-cell functions.^3,4^ Global incidence of T2DM has markedly risen in the past three decades, affecting approximately 422 million individuals worldwide, with a majority residing in low- to middle-income countries. DM, which directly contributes to about 1.5 million deaths annually, has continued to increase steadily over recent decades in both prevalence and the number of cases.^5^ Antidiabetic agents have been identified that control blood glucose levels generally act by enhancing insulin secretion (sulfonylureas and meglitinides) from pancreatic beta cells, improving insulin sensitivity to organ receptors (biguanides and thiazolidinediones), reducing carbohydrate absorption in the gastrointestinal tract (α-glucosidase inhibitors), preventing glucose reabsorption and ensuring glucose excretion in the urine (SGLT-2 inhibitors), or stimulating glucose-dependent insulin secretion and suppressing glucagon secretion in the pancreas (GLP-1 agonists and DPP4 inhibitors).^6−8^ However, intensive studies are being carried out to discover new antidiabetic drugs with increased selectivity and specificity because some of these drugs lose their effectiveness over time and have undesirable side effects in some patients.^9^

A major scientific focus for hyperglycemic control in diabetic patients is the design of α-amylase and α-glucosidase inhibitors to reduce intestinal glucose absorption.^10^ α-amylase, secreted from the salivary glands and pancreas, plays a role in the breakdown of carbohydrates such as starch and glycogen into oligosaccharides. Similarly, α-glucosidase is another class of enzyme that catalyzes the hydrolysis of the glycosidic bonds of carbohydrates and then breaks them down into monosaccharides.^11^ Commercially available α-amylase and α-glucosidase inhibitors such as acarbose, voglibose, and miglitol, which reduce glucose absorption by delaying the digestion of carbohydrates and thus causing a decrease in blood glucose levels, have some disadvantages of containing sugar units, having very long synthesis steps, and also causing serious gastrointestinal side effects such as bloating, abdominal pain, nausea, constipation, and diarrhea in clinical use. Additionally, while these drugs weakly inhibit α-amylase enzymes, they essentially inhibit α-glucosidase enzymes.^12,13^ Therefore, the discovery of small molecules with strong dual inhibitory activities, minimal adverse drug reactions, and better efficacy has attracted great interest in recent years due to the relationship between α-amylase and α-glucosidase.

On the other hand, as a result of the metabolic abnormalities observed in the onset and progression of diabetes, neoplastic transformation occurs with the increase in supraphysiological concentrations of both insulin and glucose to which body tissues are exposed, creating a powerful growth factor and energy source necessary for cancer progression, especially in individuals with T2DM.^14^ Recent studies have shown that high glucose levels (hyperglycemia) are responsible for the induction of oxidative stress and DNA damage that can trigger the early stages of tumorigenesis and alter the expression of genes involved in proliferation, migration and cell adhesion.^15^ Furthermore, endogenous hyperinsulinemia, which is mainly observed in T2DM patients in response to decreased insulin sensitivity of peripheral tissues, may stimulate the proliferation directly through the insulin receptor and indirectly through insulin-like growth factor 1 and survival of cancer cells as well as promote metastasis.^16^

Insulin, a peptide hormone secreted by pancreatic islet beta cells, plays a pivotal role in the homeostatic regulation of glucose and energy metabolism. In response to elevated glucose levels, insulin binds to cell membrane insulin receptors, facilitating the uptake of glucose, proteins, and various other molecules. Beyond its essential role in human growth and development, insulin exhibits antiapoptotic properties and functions as a growth factor by promoting mitosis through the Akt signaling pathway.^17^ Numerous studies have reported a positive correlation between elevated serum insulin levels and the development of various cancers.^18−22^

**Figure 1:**
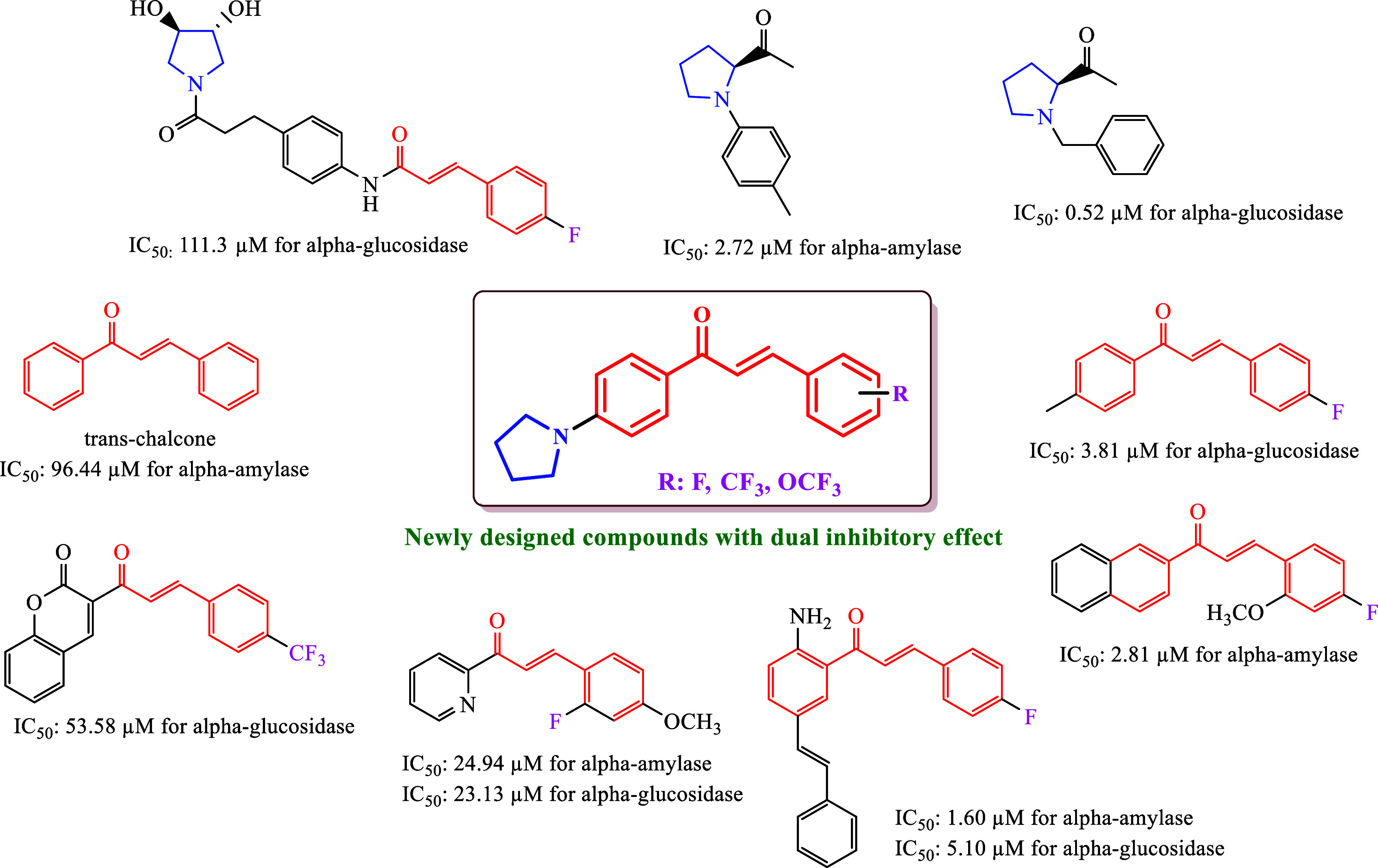
Design strategy.

*Trans*-chalcones (1,3-diaryl-2-propen-1-ones) are one of the flavonoid subtypes with an α,β-unsaturated carbonyl structure that attract great attention in medicinal chemistry and have a rich spectrum of pharmacological activities such as antidiabetic, antioxidant, antiviral, antiinflammatory, antimicrobial, anticancer, anticholinesterase, antituberculosis, antidepressant, etc.^23−33^ In addition, the pyrrolidine ring, which is among the most common five-membered nonaromatic nitrogen heterocyclic systems that are part of many drugs approved by the FDA, is one of the most preferred fragments in drug discovery studies.^34,35^ Based on the idea that the presence of more than one pharmacophore group in the same molecule may create the expected pharmacological effect, the *trans*-chalcone skeleton with α-amylase inhibition activity was selected as the main structural moiety in this study. Also, new molecules were designed by combining pyrrolidine ring and fluorine-containing substituents at different positions (Figure 1).^36−43^ Therefore, a series of *trans*-chalcones with pyrrolidine rings were synthesized to obtain new biologically active hybrid compounds that may have dual inhibitory effects against α-amylase and α-glycosidase as well as anticancer activity. Also, novel chalcone derivatives were tested for their *in vitro* antidiabetic against α-amylase and α-glucosidase and anticancer activities against nonsmall lung cancer (A549) and bronchial epithelial normal (BEAS-2B) cell lines and molecular docking studies, and their absorption, distribution, metabolism, and excretion (ADME) profiles were carried out.

## 2 Experimental Section

### 2.1 Materials and Methods

All chemicals and solvents were of analytical grade, purchased from Acros, Alfa Aesar, Sigma-Aldrich and Merck. Chemical reactions were monitored by using thin-layer chromatography (TLC, Merck 60 F_254_). Melting points were determined by the SMP20 melting point apparatus and were uncorrected. FTIR spectra were recorded on the PerkinElmer Frontier spectrometer by attenuated total reflectance apparatus (Waltham, Massachusetts, USA). ^1^H-and ^13^C NMR spectra were recorded on an Agilent Technologies instrument with 400 MHz NMR (Agilent, USA). Mass analyses were performed by the ionization method on LC–MS/MS Agilent Technologies 1260 Infinity II, 6460 Triple Quad Mass Spectrometer device. Antidiabetic inhibitory activities were carried out on a 96-well microplate reader, SpectraMax 340PC^384^, Molecular Devices (USA). Cytotoxic activity was measured with an Epoch microplate spectrophotometer (BioTek Instruments, Winooska, VT). Spectroscopic data of compounds **1–13** are given in the Supporting Information.

### 2.2 General Procedure of Compounds **1**–**13**

#### 2.2.1 Synthesis of Compounds **1**–**13** Was Carried Out according to the Previously Documented Method.^44,45^

##### 2.2.1.1 (*E*)-3-Phenyl-1-(4-(pyrrolidin-1-yl)phenyl)prop-2-en-1-one (**1**)^46^

Yellow solid; yield: 35%, mp 216–217 °C. FTIR νmax (cm^–1^): 3058, 2970 (Ar–CH); 1641 (C=O); 1609, 1597, 1578, 1565, 1539 (C=C). LC–MS (*m*/*z*): C_19_H_19_NO (277.15) 278.10 [M + H]^+^.

##### 2.2.1.2 (*E*)-3-(2-fluorophenyl)-1-(4-(pyrrolidin-1-yl)phenyl)prop-2-en-1-one (**2**)

Orange solid, yield: 57%, mp 154–155 °C. FTIR νmax (cm^–1^): 2957, 2846 (C–H); 1643 (C=O); 1606, 1568, 1538, 1482 (C=C); 1226 (C–F). ^1^H NMR (400 MHz) (DMSO-*d*_6_/TMS): δ ppm: 1.99 (t, 4H, *J*_1_ = 6.40, *J*_2_ = 6.80 Hz); 3.36 (t, 4H, *J*_1_ = 4.00, *J*_2_ = 6.40 Hz); 6.63 (d, 2H, *J* = 8.80 Hz); 7.29–7.34 (m, 2H); 7.47–7.52 (m, 1H); 7.76 (d, 1H, *J* = 16.00 Hz); 7.97 (d, 1H, *J*: 15.20 Hz); 8.04 (d, 2H, *J* = 8.40 Hz); 8.11 (t, 1H, *J*_1_: 8.00, *J*_2_: 7.60 Hz). ^13^C NMR (100 MHz) (DMSO-*d*_6_/TMS): δ ppm: 25.41, 47.81, 111.54, 116.59, 123.30, 124.95, 125.39, 129.39, 131.43, 132.48, 133.13, 151.43, 159.98, 162.47, 186.08. LC–MS (*m*/*z*): C_19_H_18_FNO (295.14) 296.00 [M + H]^+^.

#### 2.2.2 (*E*)-3-(3-fluorophenyl)-1-(4-(pyrrolidin-1-yl)phenyl)prop-2-en-1-one (**3**)

Orange solid, yield: 73%, mp 196–1988 °C. FTIR νmax (cm^–1^): 2965, 2853 (C–H); 1644 (C=O); 1603, 1581, 1567, 1539 (C=C); 1249 (C–F). ^1^H NMR (400 MHz) (DMSO-*d*_6_/TMS): δ ppm: 1.99 (t, 4H, *J*_1_ = 6.40, *J*_2_ = 6.80 Hz); 3.36 (t, 4H, *J*_1_ = 4.40, *J*_2_ = 6.40 Hz); 6.62 (d, 2H, *J* = 8.80, Hz); 7.26 (t, 1H, *J*_1_ = 8.80, *J*_2_ = 8.40 Hz); 7.46–7.51 (m, 1H); 7.63 (d, 1H, *J* = 15.20 Hz); 7.66 (d, 1H, *J* = 8.00), 7.83 (d, 1H, *J* = 8.40); 7.99 (d, 1H, *J* = 15.60 Hz); 8.07 (d, 2H, *J* = 9.20 Hz). ^13^C NMR (100 MHz) (DMSO-*d*_6_/TMS): δ ppm: 25.41, 47.80, 111.47, 114.93, 117.20, 124.41, 125.10, 125.69, 131.49, 138.32, 140.47, 151.39, 161.80, 164.22, 186.23. LC–MS (*m*/*z*): C_19_H_18_FNO (295.14) 296.10 [M + H]^+^.

##### 2.2.2.1 (*E*)-3-(4-fluorophenyl)-1-(4-(pyrrolidin-1-yl)phenyl)prop-2-en-1-one (**4**)

Yellow solid, yield: 60%, mp 207–210 °C. FTIR νmax (cm^–1^): 2975, 2854 (C–H); 1646 (C=O); 1590, 1578, 1541, 1508 (C=C); 1226 (C–F)·^1^H NMR (400 MHz) (DMSO-*d*_6_/TMS): δ ppm: 2.00 (t, 4H, *J*_1_ = 6.40, *J*_2_ = 6.80 Hz); 3.37 (t, 4H, *J*_1_ = 6.40, *J*_2_ = 6.80 Hz); 6.62 (d, 2H, *J* = 8.40 Hz); 7.29 (t, 2H, *J*_1_ = 8.40, *J*_2_ = 8.80 Hz); 7.63 (d, 1H, *J* = 15.60 Hz); 7.89 (d, 1H, *J* = 15.60 Hz); 7.92–7.96 (m, 2H); 8.06 (d, 2H, *J*: 8.40 Hz). ^13^C NMR (100 MHz) (DMSO-*d*_6_/TMS): δ ppm: 25.42, 47.80, 111.46, 116.40, 121.38, 125.04, 126.34, 128.10, 131.38, 142.05, 145.70, 153.78, 159.07, 162.95, 180.60. LC–MS (*m*/*z*): C_19_H_18_FNO (295.14) 296.10 [M + H]^+^.

##### 2.2.2.2 (*E*)-1-(4-(pyrrolidin-1-yl)phenyl)-3-(2-(trifluoromethyl)phenyl)prop-2-en-1-one (**5**)

Yellow solid, yield: 70%, mp 163–164 °C. FTIR νmax (cm^–1^): 2959, 2847 (C–H); 1644 (C=O); 1605, 1582, 1570, 1541 (C=C); 1228 (C–F). ^1^H NMR (400 MHz) (DMSO-*d*_6_/TMS): δ ppm: 1.98 (t, 4H, *J*_1_ = 6.80, *J*_2_ = 6.80 Hz); 3.36 (t, 4H, *J*_1_ = 4.00, *J*_2_ = 6.40 Hz); 6.62 (d, 2H, *J* = 8.80 Hz); 7.64 (t, 1H, *J*_1_ = 7.60, *J*_2_ = 7.60 Hz); 7.78 (t, 1H, *J*_1_ = 7.60, *J*_2_ = 7.60 Hz); 7.83 (d, 1H, *J* = 8.00 Hz); 7.90 (d, 1H, *J* = 15.20 Hz); 8.00 (d, 1H, *J* = 15.20 Hz); 8.07 (d, 2H, *J* = 8.40 Hz); 8.31 (d, 1H, *J* = 7.60 Hz). ^13^C NMR (100 MHz) (DMSO-*d*_6_/TMS): δ ppm: 25.40, 47.81, 111.55, 124.77, 126.10, 126.58, 127.15, 127.57, 129.08, 130.40, 131.61, 133.37, 133.97, 135.95, 151.52, 185.82. LC–MS (*m*/*z*): C_20_H_18_F_3_NO (345.13) 346.10 [M + H]^+^.

##### 2.2.2.3 (*E*)-1-(4-(pyrrolidin-1-yl)phenyl)-3-(3-(trifluoromethyl)phenyl)prop-2-en-1-one (**6**)

Orange solid, yield: 59%, mp 184–186 °C. FTIR νmax (cm^–1^): 2979, 2852 (C–H); 1644 (C=O); 1603, 1568, 1538, 1494 (C=C); 1227 (C–F). ^1^H NMR (400 MHz) (DMSO-*d*_6_/TMS): δ ppm: 1.99 (t, 4H, *J*_1_ = 6.40, *J*_2_ = 6.80 Hz); 3.37 (t, 4H, *J*_1_ = 7.60, *J*_2_ = 6.80 Hz); 6.62 (d, 2H, *J* = 9.20 Hz); 7.66–7.78 (m, 3H); 8.12–8.08 (m, 3H); 8.15 (d, 1H, *J* = 8.00 Hz); 8.30 (s, 1H). ^13^C NMR (100 MHz) (DMSO-*d*_6_/TMS): δ ppm: 25.41, 47.81, 111.46, 124.96, 125.07, 125.27, 126.56, 130.09, 130.33, 130.41, 131.57, 132.99, 136.87, 140.10, 151.42, 186.20. LC–MS (*m*/*z*): C_20_H_18_F_3_NO (345.13) 346.10 [M + H]^+^.

##### 2.2.2.4 (*E*)-1-(4-(pyrrolidin-1-yl)phenyl)-3-(4-(trifluoromethyl)phenyl)prop-2-en-1-one (**7**)

Light green solid, yield: 53%, mp 209–210 °C. FTIR νmax (cm^–1^): 2977, 2855 (C–H); 1645 (C=O); 1590, 1577, 1541, 1507 (C=C); 1227 (C–F). ^1^H NMR (400 MHz) (DMSO-*d*_6_/TMS): δ ppm: 2.00 (t, 4H, *J*_1_ = 6.80, *J*_2_ = 6.80 Hz); 3.36 (t, 4H, *J*_1_ = 6.40, *J*_2_ = 6.80 Hz); 6.62 (d, 2H, *J* = 8.80 Hz); 7.27 (t, 2H, *J*_1_ = 7.26, *J*_2_ = 8.80 Hz); 7.63 (d, 1H, *J* = 15.60 Hz); 7.84 (d, 1H, *J* = 15.20 Hz); 7.91 (t, 2H, *J*_1_ = 5.60, *J*_2_ = 8.40 Hz); 8.04 (d, 2H, *J* = 8.40 Hz). ^13^C NMR (100 MHz) (DMSO-*d*_6_/TMS): δ ppm: 25.42, 47.80, 111.45, 116.18, 116.40, 122.83, 125.21, 131.23, 131.38, 132.35, 140.67, 151.31, 162.29, 186.31. LC–MS (*m*/*z*): C_20_H_18_F_3_NO (345.13) 346.10 [M + H]^+^.

#### 2.2.3 (*E*)-1-(4-(pyrrolidin-1-yl)phenyl)-3-(3-(trifluoromethoxy)phenyl)prop-2-en-1-one (**8**)

Mustard yellow solid, yield: 35%, mp 141–143 °C. FTIR νmax (cm^–1^): 2968, 2846 (C–H); 1644 (C=O); 1598, 1572, 1540, 1478 (C=C); 1240 (C–F). ^1^H NMR (400 MHz) (DMSO-*d*_6_/TMS): δ ppm: 2.00 (t, 4H, *J*_1_ = 8.00, *J*_2_ = 6.80 Hz); 3.37 (t, 4H, *J*_1_ = 2.80, *J*_2_ = 6.80 Hz); 6.56 (d, 2H, *J* = 8.80 Hz); 7.41 (d, 1H, *J* = 8.00 Hz); 7.58 (t, 1H, *J*_1_ = 8.00, *J*_2_ = 8.00 Hz); 7.65 (d, 1H, *J* = 15.60 Hz); 7.79 (d, 2H, *J* = 8.80 Hz); 7.88 (d, 1H, *J* = 7.60 Hz); 7.96 (s, 1H); 8.03 (d, 1H, *J* = 15.60 Hz). ^13^C NMR (100 MHz) (DMSO-*d*_6_/TMS): δ ppm: 25.41, 47.73, 121.13, 122.54, 124.71, 124.85, 128.38, 130.80, 131.21, 131.54, 138.24, 140.06, 149.35, 151.15, 186.17, 195.53. LC–MS (*m*/*z*): C_20_H_18_F_3_NO2 (361.13) 362.00 [M + H]^+^.

##### 2.2.3.1 (*E*)-1-(4-(pyrrolidin-1-yl)phenyl)-3-(4-(trifluoromethoxy)phenyl)prop-2-en-1-one (**9**)

Dark yellow solid, yield: %56, mp 151–153 °C. FTIR νmax (cm^–1^): 2973, 2848 (C–H); 1644 (C=O); 1603, 1569, 1540, 1509 (C=C); 1245 (C–F). ^1^H NMR (400 MHz) (DMSO-*d*_6_/TMS): δ ppm: 1.98 (t, 4H, *J*_1_ = 6.40, *J*_2_ = 6.80 Hz); 3.35 (t, 4H, *J*_1_ = 4.80, *J*_2_ = 8.00 Hz); 6.62 (d, 2H, *J* = 8.80 Hz); 7.44 (d, 2H, *J* = 8.40 Hz); 7.65 (d, 1H, *J* = 15.20 Hz); 7.96 (d, 1H, *J* = 15.60 Hz); 8.01 (d, 2H, *J* = 8.80 Hz); 8.06 (d, 2H, *J* = 8.80 Hz). ^13^C NMR (100 MHz) (DMSO-*d*_6_/TMS): δ ppm: 25.41, 47.80, 111.47, 121.73, 121.79, 124.16, 125.10, 130.90, 131.44, 135.06, 140.11, 149.61, 151.37, 186.22. LC–MS (*m*/*z*): C_20_H_18_F_3_NO2 (361.13) 362.00 [M + H]^+^.

##### 2.2.3.2 (*E*)-3-(3,5-bis(trifluoromethyl)phenyl)-1-(4-(pyrrolidin-1-yl)phenyl)prop-2-en-1-one (**10**)

Yellow solid, yield: 30%, mp 200–202 °C. FTIR νmax (cm^–1^): 2962, 2848 (C–H); 1647 (C=O); 1606, 1577, 1541, 1483 (C=C); 1238 (C–F). ^1^H NMR (400 MHz) (DMSO-*d*_6_/TMS): δ ppm: 2.00 (t, 4H, *J*_1_ = 6.80, *J*_2_ = 6.40 Hz); 3.38 (t, 4H, *J*_1_ = 6.80, *J*_2_ = 6.40 Hz); 6.63 (d, 2H, *J* = 8.80 Hz); 7.78 (d, 1H, *J* = 15.60 Hz); 8.07 (s, 1H); 8.11 (d, 2H, *J* = 8.40 Hz); 8.24 (d, 1H, *J* = 15.60 Hz); 8.60 (s, 1H). ^13^C NMR (100 MHz) (DMSO-*d*_6_/TMS): δ ppm: 25.40, 47.82, 111.46, 122.43, 122.98, 126.88, 129.50, 130.80, 131.13, 131.73, 138.48, 138.63, 151.51, 185.98. LC–MS (*m*/*z*): C_21_H_17_F_6_NO (413.12) 414.14 [M + H]^+^.

##### 2.2.3.3 (*E*)-3-(2-fluoro-3-(trifluoromethyl)phenyl)-1-(4-(pyrrolidin-1-yl)phenyl)prop-2-en-1-one (**11**)

Orange solid, yield: 38%, mp 194–196 °C. FTIR νmax (cm^–1^): 2978, 2858 (C–H); 1644 (C=O); 1607, 1573, 1543, 1463 (C=C); 1240 (C–F). ^1^H NMR (400 MHz) (DMSO-*d*_6_/TMS): δ ppm: 1.99 (t, 4H, *J*_1_ = 6.40, *J*_2_ = 6.80 Hz); 3.37 (t, 4H, *J*_1_ = 6.40, *J*_2_ = 6.80 Hz); 6.64 (d, 2H, *J* = 8.80 Hz); 7.52 (t, 1H, *J*_1_ = 8.00, *J*_2_ = 8.00 Hz); 7.75 (d, 1H, *J* = 15.60 Hz); 7.85 (t, 1H, *J*_1_ = 7.20, *J*_2_ = 7.60 Hz); 8.06 (d, 2H, *J* = 8.80 Hz); 8.08 (d, 1H, *J* = 15.60 Hz); 8.46 (t, 1H, *J*_1_ = 8.80, *J*_2_ = 7.20 Hz). ^13^C NMR (100 MHz) (DMSO-*d*_6_/TMS): δ ppm: 25.41, 47.73, 121.13, 122.54, 124.71, 124.85, 128.38, 130.80, 131.21, 131.54, 138.24, 140.06, 149.35, 151.15, 186.17, 195.53. LC–MS (*m*/*z*): C_20_H_17_F_4_NO (363.12) 364.10 [M + H]^+^.

##### 2.2.3.4 (*E*)-3-(2-fluoro-4-(trifluoromethyl)phenyl)-1-(4-(pyrrolidin-1-yl)phenyl)prop-2-en-1-one (**12**)

Mustard yellow solid, yield: 46%, mp 145–149 °C. FTIR νmax (cm^–1^): 2962, 2866 (C–H); 1644 (C=O); 1604, 1573, 1542, 1528 (C=C); 1239 (C–F). ^1^H NMR (400 MHz) (DMSO-*d*_6_/TMS): δ ppm: 2.00 (t, 4H, *J*_1_ = 6.80, *J*_2_ = 6.40 Hz); 3.37 (t, 4H, *J*_1_ = 3.20, *J*_2_ = 6.00 Hz); 6.63 (d, 2H, *J* = 8.80 Hz); 7.37 (s, 1H); 7.68 (d, 1H, *J* = 8.40 Hz); 7.73 (d, 1H, *J* = 16.00 Hz); 7.79 (d, 1H, *J* = 10.40 Hz); 8.06 (d, 2H, *J* = 8.80 Hz); 8.11 (d, 1H, *J* = 15.60 Hz); 8.34 (d, 1H, *J* = 8.00 Hz). ^13^C NMR (100 MHz) (DMSO-*d*_6_/TMS): δ ppm: 25.40, 47.82, 111.58, 122.10, 124.75, 125.42, 127.45, 127.74, 130.52, 131.35, 131.60, 134.60, 151.55, 159.42, 161.93, 185.72. LC–MS (*m*/*z*): C_20_H_17_F_4_NO (363.12) 364.10 [M + H]^+^.

##### 2.2.3.5 (*E*)-3-(4-fluoro-3-(trifluoromethyl)phenyl)-1-(4-(pyrrolidin-1-yl)phenyl)prop-2-en-1-one (**13**)

Yellow solid, yield: %75, mp 216–218 °C. FTIR νmax (cm^–1^): 2961, 2842 (C–H); 1644 (C=O); 1597, 1570, 1541, 1504 (C=C); 1226 (C–F). ^1^H NMR (400 MHz) (DMSO-*d*_6_/TMS): δ ppm: 1.99 (t, 4H, *J*_1_ = 3.60, *J*_2_ = 6.40 Hz); 3.36 (t, 4H, *J*_1_ = 6.00, *J*_2_ = 7.20 Hz); 6.61 (d, 2H, *J* = 8.80 Hz); 7.34 (d, 1H, *J* = 9.60 Hz); 7.66 (d, 1H, *J* = 15.60 Hz); 7.91 (d, 1H, *J* = 15.20 Hz); 8.06–8.13 (m, 4H). ^13^C NMR (100 MHz) (DMSO-*d*_6_/TMS): δ ppm: 25.42, 47.80, 78.09, 111.16, 111.42, 113.76, 122.31, 125.27, 127.56, 128.12, 131.44, 131.58, 135.02, 140.38, 151.29, 158.57, 186.30. LC–MS (*m*/*z*): C_20_H_17_F_4_NO (363.12) 364.10 [M + H]^+^.

### 2.3 Determination of Antidiabetic Activity

#### 2.3.1 Determination of α-Amylase Inhibitory Activity of Compounds **1–13**

The α-amylase inhibitory activity of compounds **1**–**13** was evaluated using a spectrophotometric method, with slight modifications from previously established protocols.^47^ Briefly, 25 μL of the sample solution at various concentrations was mixed with 50 μL of an α-amylase solution (0.1 U/mL) in a phosphate buffer (20 mM, pH 6.9, prepared with 6 mM NaCl) in a 96-well microplate. This mixture was preincubated at 37 °C for 10 min. Following the preincubation, 50 μL of a starch solution (0.05%) was added, and the reaction mixture was incubated for another 10 min at 37 °C. The reaction was then terminated by adding 25 μL of HCl (0.1 M), followed by the addition of 100 μL of Lugol’s solution for monitoring. Absorbance was measured at 565 nm using a 96-well microplate reader.

#### 2.3.2 Determination of α-Glucosidase Inhibitory Activity of Compounds **1–13**

The α-glucosidase inhibitory activity of compounds **1**–**13** was determined using a spectrophotometric method, with slight modifications from previously reported methods.^48^ Specifically, 50 μL of phosphate buffer (10 mM, pH 6.9), 25 μL of *p*-nitrophenyl-α-d-glucopyranoside in phosphate buffer (10 mM, pH 6.9), 10 μL of sample solution, and 25 μL of α-glucosidase (0.1 U/mL) in phosphate buffer (10 mM, pH 6.0) were combined in a 96-well microplate. After 20 min of incubation at 37 °C, 90 μL of Na_2_CO_3_ (100 mM) was added to each well to halt the enzymatic reaction. The absorbance was measured at 400 nm by using a 96-well microplate reader.

### 2.4 Cytotoxic Activity Assay

#### 2.4.1 Cell Culture Condition

In this research, the nonsmall lung cancer (A549) and bronchial epithelial normal (BEAS-2B) cells were used for cytotoxicity experiments. All of the cells were cultured in Dulbecco’s Modified Eagle Medium (Gibco, USA) cell medium containing 10% fetal calf serum (FBS; Gibco, USA) and 1% penicillin/streptomycin (Gibco, USA) in an incubator at 37 °C and 5% CO_2_.^49,50^

#### 2.4.2 Chemical Exposure

In a 96-well cell culture plate, cells were seeded at a density of 6 × 104 cells/mL and observed to be cultured for 24 h. The cells must have 70% confluence before chemical exposure. After 70% confluency incubation, the cells were treated with different concentrations of compounds for 24 h. All chemicals were dissolved in DMSO. Commercially purchased cisplatin (Koçak Farma, Türkiye) was used as a positive control and tested at the same concentrations. The amount of DMSO contained in the chemical at the highest concentration was used as a negative control.

#### 2.4.3 Cell Viability Assay

Cell viability was determined by 3-[4,5-dimethylthiazol-2-yl]-2,5-diphenyltetrazolium bromide (MTT) assay. This method is specifically used to evaluate live cells. The succinate dehydrogenase system of active mitochondria reduces the tetrazolium salt MTT to formazan.^51^ For the MTT assay, MTT solution (mg/mL) was added into the cells (200 μL/well) and incubated for 1 h at 37 °C. Absorbance was measured at 570 nm using an Epoch microplate spectrophotometer (BioTek Instruments, Winooska, VT).^52^

### 2.5 In Silico Studies

#### 2.5.1 Molecular Docking Simulations

Molecular docking is a computational technique widely employed in drug discovery and structural biology to predict the binding mode and affinity between a small molecule (ligand) and a target macromolecule (receptor), typically a protein. This technique plays a crucial role in rational drug design, lead optimization, and virtual screening.^53,54^ The 3D crystal structures of the targeted enzymes were obtained from the Protein Data Bank Web site (http://www.rcsb.org/pdb) using their respective PDB IDs: 4W93 for α-amylase and 5NN4 for α-glucosidase. The preparation steps were applied to these crystal structures, including the removal of water and ion molecules, and the addition of appropriate hydrogen atoms under physiological pH conditions (pH = 7) using the APBS-PDB 2PQR software.^55^ The grid box for docking was determined based on the binding site location of the crystallized ligands within the enzymes. The docking procedure followed standard protocols, employing a rigid protein and a flexible ligand, and was executed using the Lamarckian Genetic algorithm with 100 generations with AutoDock 4.2.^56^ Throughout the analysis, predictions were made for binding free energy (Δ*G*) and inhibitory constant (*K*_*i*_) values for all of the compounds.

#### 2.5.2 Molecular Dynamic Simulation

A molecular dynamics (MD) simulation with a duration of 100 ns was conducted using the Desmond software to assess the binding stability of the most potent compound 3 and the reference compound, acarbose, with the α-amylase and α-glucosidase proteins.^57^ Simulation parameters were configured through the Maestro 13.8 graphical interface integrated with Desmond. The protein–ligand complexes were positioned within an orthorhombic simulation box and solvated using the TIP3P water model.^58^ Na^+^ and Cl^–^ ions were introduced at a physiological concentration of 0.15 M to neutralize the system. The OPLS4 force field was applied to describe the molecular interactions within the system. The simulations were executed under the *NPT* ensemble conditions, maintaining a temperature of 300 K and a pressure of 1.01325 bar while keeping the particle count constant. Temperature regulation was achieved using the Nosé–Hoover thermostat, and pressure stability was ensured by the Martyna–Tobias–Klein barostat. Long-range electrostatic interactions were computed by using the Particle Mesh Ewald method. In contrast, short-range electrostatic and van der Waals forces were confined to a cutoff distance of 9.0 Å, ensuring precise modeling of intermolecular interactions.^59^

Detailed trajectory analyses were carried out to examine the dynamic properties and stability of the protein–ligand complexes. Structural parameters such as root mean square deviation (RMSD) and root mean square fluctuation (RMSF) were quantified alongside evaluating binding interactions, including hydrogen bonds and hydrophobic contacts. Insights into the protein and ligand interactions highlighted crucial noncovalent interactions, including hydrogen bonds, hydrophobic contacts, ionic interactions, and water-mediated bridges. These findings enhance understanding of molecular mechanisms governing protein–ligand interactions and offer valuable guidance for structure-based drug design.

### 2.6 Statistical Analysis

All biological activity data were taken in three parallel measurements for four different concentrations of each synthesis sample. The results of the biological activity analyses are presented as the IC_50_ values. While antidiabetic inhibition activities were recorded as mean ± standard error of the mean (SEM) *p* < 0.01, cytotoxic activity was analyzed with an analysis of variance test (prism 8 software) *p* < 0.05.

## 3 Result and Discussion

### 3.1 Chemistry

**Scheme 1:**
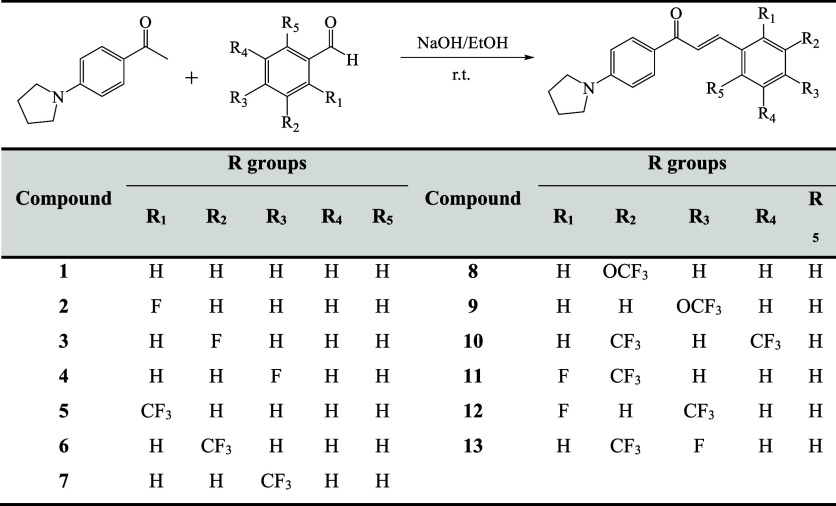
Synthetic Pathway of (*E*)-3-(Substitutedphenyl)-1-(4-(Pyrrolidin-1-yl)phenyl)prop-2-en-1-Ones (**1**–**13**)

(*E*)-3-(substitutedphenyl)-1-(4-(pyrrolidin-1-yl)phenyl)prop-2-en-1-ones (**1**–**13**) were synthesized according to Claisen Schmidt condensation and 4′-(1-pyrrolidino)acetophenone were treated with the substituted benzaldehydes carrying F, CF_3_, OCF_3_ groups located in different positions. Chalcones containing pyrrolidine rings were synthesized in 75–38% yield. The synthesis pathway and R groups of (*E*)-3-(substitutedphenyl)-1-(4-(pyrrolidin-1-yl)phenyl)prop-2-en-1-one (**1**–**13**) are shown in Scheme 1. Chalcones containing pyrrolidine rings were synthesized in 38–75% yield.

The FT-IR spectra of compounds **1**–**13**, aliphatic C–H stretching bands were detected in the range of 2979–2842 cm^–1^, C=O stretching bands were observed at 1643–1647 cm^–1^; C–F stretching bands were seen at 1249–1226 and the C=C stretching bands were determined at 1607–1463 cm^–1^.

In the ^1^H NMR spectra, H_α_ and H_β_ protons of compounds **1**–**13** were resonated at 7.63–7.90 ppm and 7.84–8.11 ppm, respectively, which was the most important evidence for α,β-unsaturated chalcone compounds. Additionally, the interaction constants of these protons were in the range of 15.20–16.00 Hz, confirming that the chalcone derivatives were in the *E* configuration. The other protons were detected in the expected regions. In the ^13^C NMR spectra, the C=O peaks of all compounds resonated in the range of 185.72–186.31 ppm.

### 3.2 Pharmacological Activities

#### 3.2.1 Antidiabetic Activity

The antidiabetic activity of pyrrolidine-chalcone hybrids **1**–**13** against α-amylase and α-glucosidase was determined in the *in vitro* enzyme assay using acarbose as a reference standard. The antidiabetic activity results are given in Table 1. Generally, the IC_50_ values of compounds **1**–**13** were found to be below 50 μM for compounds **1**, **3**, **4**, **7**, **8**, **9**, **10**, and **11** in the α-amylase inhibition assay and compounds **3**, **7**, and **10** in the α-glucosidase inhibition assay. All compounds, except compound **5**, were found to be more active than acarbose in the α-amylase inhibition assay. Moreover, compounds **3** (IC_50_: 14.61 ± 0.12 μM) and **8** (IC_50_: 18.10 ± 0.34 μM) were found to exhibit great α-amylase inhibitory effect. Compounds **3**, **7**, **10**, **1**, **6**, **4**, **9**, and **12** were found to have a better inhibitory effect against α-glucosidase than the positive standard acarbose, respectively. Among the series of chalcone, compounds **3** (IC_50_: 25.38 ± 2.09 μM) and **10** (IC_50_: 44.21 ± 2.42 μM) were found to be the most active inhibitors.

**Table 1:** Antidiabetic Inhibition and Cytotoxic Activities of Chalcones (**1–13**)^a^

compound	antidiabetic inhibition activity IC_50_ (μM)^c^		cytotoxic activity^d^ IC_50_ (μM)	
	α-amylase	α-lucosidase	A549	BEAS-2B
**1**	**21.78 ± 1.59**	**50.21 ± 2.32**	>100	>100
**2**	**51.96 ± 0.12**	188.65 ± 1.94	>100	>100
**3**	**14.61 ± 0.12**	**25.38 ± 2.09**	>100	>100
**4**	**30.63 ± 0.25**	**58.92 ± 3.26**	>100	>100
**5**	91.71 ± 0.28	172.73 ± 3.45	**82.20**	**97.72**
**6**	**83.78 ± 0.38**	**54.06 ± 2.87**	>100	>100
**7**	**38.22 ± 0.83**	**46.50 ± 2.16**	>100	>100
**8**	**18.10 ± 0.34**	147.49 ± 0.90	**59.96**	**>100**
**9**	**39.97 ± 0.26**	**61.30 ± 2.48**	>100	>100
**10**	**39.68 ± 0.27**	**44.21 ± 2.42**	>100	>100
**11**	**22.40 ± 0.50**	131.00 ± 3.02	>100	>100
**12**	**80.11 ± 0.49**	**81.77 ± 0.85**	>100	>100
**13**	**83.80 ± 0.01**	261.50 ± 1.69	>100	>100
**acarbose**^b^	87.90 ± 0.76	124.62 ± 0.49	N.D.	N.D.
**cisplatin**^b^	N.D.	N.D.	**84.39**	>100

a Values expressed herein are mean ± SEM of three parallel measurements. *p* < 0.05.

b Reference compounds.

c All compounds for α-amylase and α-glucosidase assay in the concentration range of 50–100–200–400 μM.

d All compounds for anticancer assay in the concentration range of 250, 125, 62.5, 31.25, 15.6, and 7.8 μM.

The structure–activity relationship study with respect to glucosidase showed that replacing the 3 F substituent at the phenyl ring with 4-CF_3_ or 3,5-diCF_3_ resulted in a decrease in the inhibition activity as seen in compounds **7** and **10**. An examination of the α-amylase inhibitory activities of the compounds revealed that the IC_50_ values of compounds (**2**, **3**, and **4**) carrying the fluorine atom at different positions of the phenyl ring were exhibited notable differences. Comparative analysis indicated that the most active derivative was compound **3** carrying a fluorine atom at the third position. Additionally, this compound was the most active inhibitor against α-amylase with an IC_50_ value of 25.38 ± 2.09 μM. Compounds **5** and **6** carrying one trifluoromethyl (CF_3_) group at the ortho and meta positions of the phenyl ring showed lesser activity than compound **7** carrying same substituent at the para position. Compound **12**, which had a fluorine atom at the second position and a CF_3_ group at the fourth position, displayed weak inhibitory activity against α-amylase. The same situation was also observed in compounds **11** and **13**. The presence of a fluorine atom and a CF_3_ group at different positions of the phenyl ring reduced the inhibitory activity.

Regarding the influence of the presence of multiple fluorine-containing substituents on the benzene ring, it is known that with a single fluorine-containing substituent, the activity data *m*-*F* > unsubstituted > *p*-*F* > *o* and *m*-*F* > *m*-OCF_3_> unsubstituted > *m*-CF_3_ in α-amylase and *m*-*F* > unsubstituted > *m*-CF_3_ > *m*-OCF_3_ in α-glucosidase.

**Figure 2:**
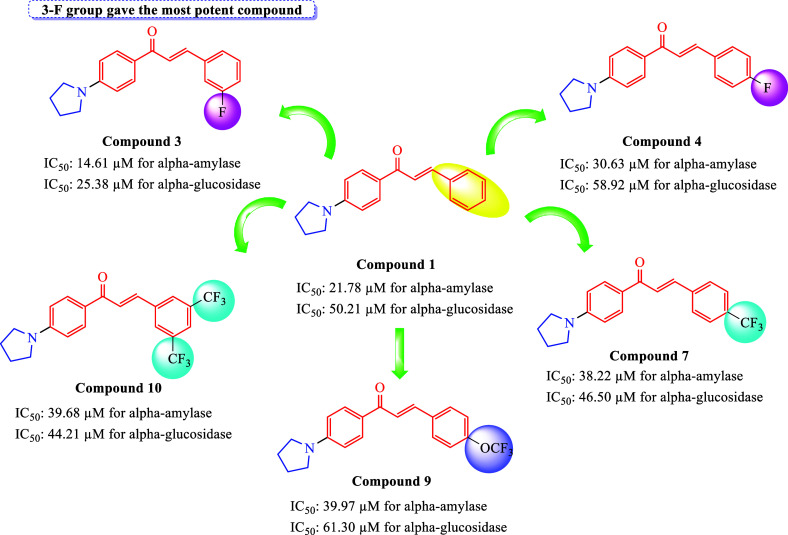
Chalcones containing pyrrolidine rings as dual inhibitors.

The α-amylase and α-glucosidase inhibitory activities of compounds **3**, **1**, **7**, **10**, **4**, and **9** were examined, and it was revealed that they exhibited a dual effect (Figure 2).

#### 3.2.2 Anticancer Activity

Cancer and diabetes are frequent diseases that have a significant global health impact. Although many different factors contribute to the development of cancer, some studies have identified an association between diabetes and cancer risk. The imbalance of blood glucose levels has been emphasized as an independent risk factor affecting cancer development and prognosis. The literature indicates that patients with diabetes have an increased probability of developing different types of cancer such as bladder, thyroid, prostate, breast, pancreas, stomach, and especially lung cancer.^60−66^ Lung cancer is a malignant neoplasm that begins from the proliferation of cells inside pulmonary tissue. NSCLC (nonsmall cell lung cancer) and SCLC (small cell lung cancer) are the two primary types of the disease. In 2020, 2.2 million new lung cancer cases and 1.8 million deaths from lung cancer were reported.^1^ Recent studies with synthetic chalcones have shown that chalcones induce apoptosis in A549 cancer cells.^67,68^ Based on these findings, this study evaluated the cytotoxic effects of the synthesized compounds on cancer cells and their toxic effects on healthy kidney cells. The MTT method was used to evaluate cell viability, and the resulting data were analyzed with Prism 8 software to calculate IC_50_ values. Cisplatin was used as a positive control in cytotoxicity tests. According to IC_50_ values in cytotoxicity experiments, among all compounds, compound **5** (IC_50_: 82.20 μM) and compound **8** (IC_50_: 59.96 μM) seem to have a high potential for treating lung cancer compared to the cisplatin (IC_50_: 84.39 μM). It was demonstrated that compounds **5** and **8** had no toxic effects on BEAS-2B cells.

According to the results of this study, both compound **5** (IC_50_: 82.20 μM) and compound **8** (IC_50_: 59.96 μM) show possible cytotoxic effects on the A549 lung cancer cell line. The most important feature of these compounds is that they are not toxic to healthy cells. Therefore, it can be claimed that compound **5**, in particular, has a high therapeutic potential in terms of its nontoxic effect on its anticancer effects (Table 1).

### 3.3 Molecular Docking Analysis

**Figure 3:**
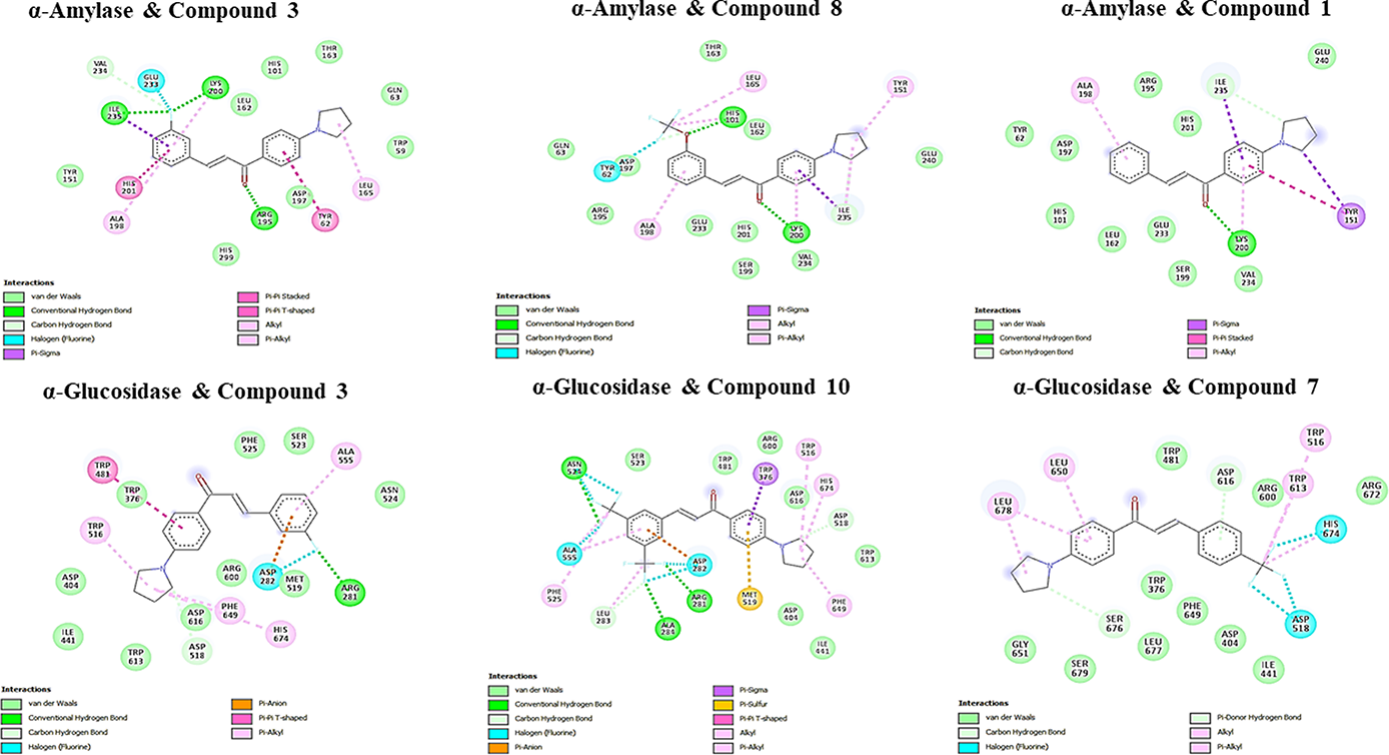
2D analysis of the lowest energy binding conformations of α-glucosidase and α-amylase with the potent compounds according to *in vitro* and *in silico* studies.

The results of the molecular docking investigation of α-amylase and α-glucosidase showed that all compounds, except compound **5**, were found to be more active than acarboseinding energy of acarbose was −6.30 and −4.66 kcal/mol, respectively. Notably, all synthesized compounds demonstrated stronger binding affinities compared to acarbose (Table 2). The *in vitro* analysis revealed that compounds **3**, **8**, and **1**, which exhibited the most potent activity among the tested compounds, displayed inhibitory activity against α-amylase at concentrations of 14.61 ± 0.12, 18.10 ± 0.34, and 21.78 ± 1.59 μM, respectively. *In silico* molecular docking analysis indicated that compounds **3**, **8**, and **1** formed various interactions with specific residues in α-amylase. These interactions included hydrogen bond interactions with residues Ile235, Lys200, Arg195, and His101, as well as pi-alkyl bond interactions with Leu165, Ala198, and Tyr151. Additionally, halogen bond interactions were observed with Tyr62 and Glu233 in α-amylase (Figure 3). These findings highlight the potential inhibitory mechanisms of compounds **3**, **8**, and **1** against α-amylase and provide insight into their structural interactions with the active site of the enzyme. This suggests that these chalcone derivatives might have improved binding abilities, indicating their potential as effective inhibitors of α-amylase.

**Table 2:** Lowest Binding Energy Values of the Synthesized Compounds (**1–13**) and Reference Compound from Each Docking Analysis in the Active Site of α-Amylase and α-Glucosidase

compound	binding energy (kcal/mol)	
	α-amylase	α-glucosidase
1	–7.17	–6.4
2	–7.16	–6.39
3	–7.25	–6.47
4	–7.21	–6.27
5	–7.16	–6.47
6	–7.49	–6.67
7	–7.4	–5.94
8	–7.53	–6.78
9	–7.65	–6.28
10	–7.69	–6.25
11	–7.34	–6.66
12	–7.43	–6.08
13	–7.52	–6.52
acarbose	–6.30	–4.66

**Figure 4:**
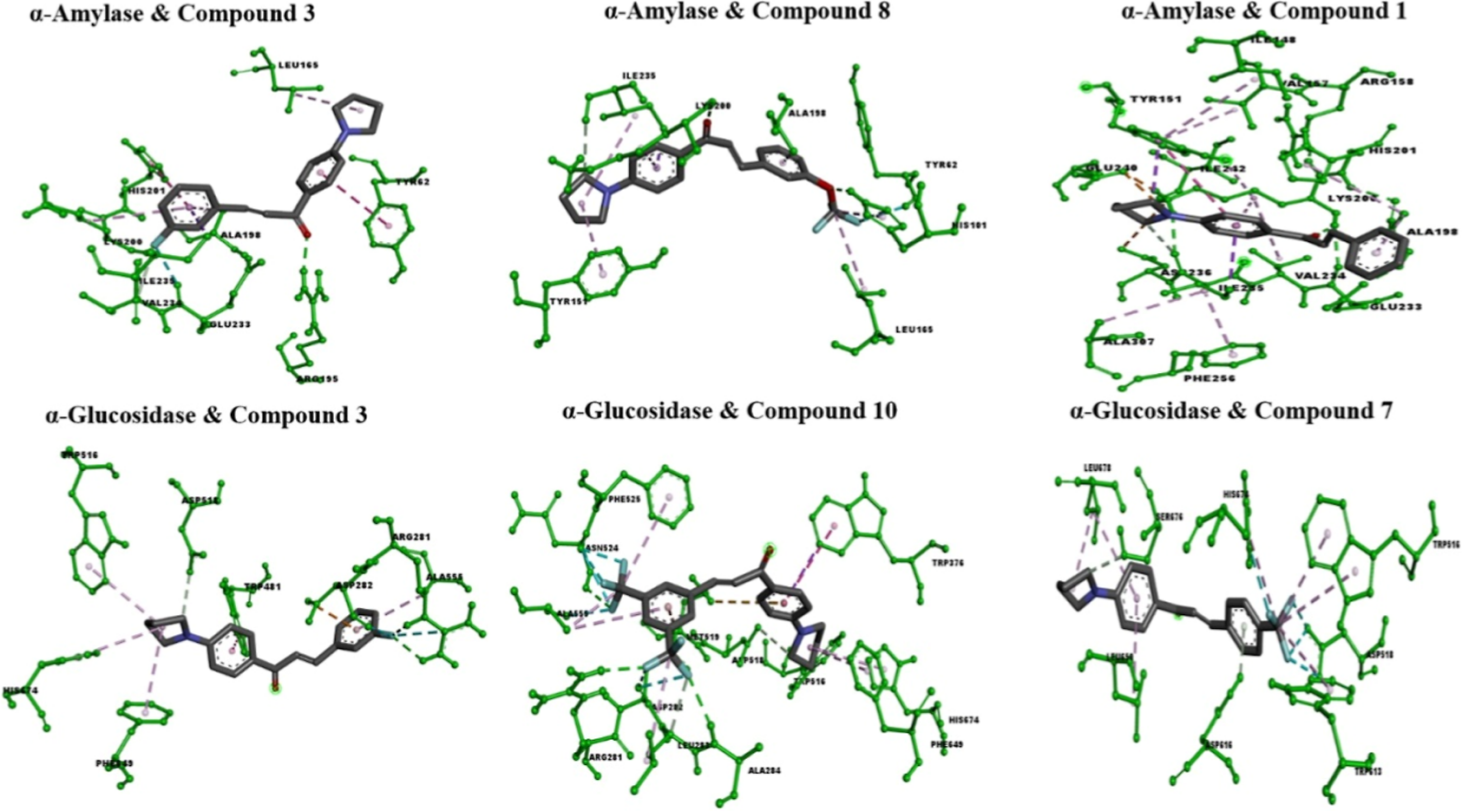
3D analysis of the lowest energy binding conformations of α-glucosidase and α-amylase with the potent compounds according to *in vitro* and *in silico* studies.

In addition, compounds **3**, **10**, and **7** exhibited significant inhibitory activity against α-glucosidase, with concentrations of 25.38 ± 2.09, 44.21 ± 2.42, and 46.50 ± 2.16 μM, respectively. Molecular docking studies indicated that these active compounds formed bonds with specific amino acids within the α-glucosidase. Specifically, they established hydrogen bond interactions with Arg281, Ala284, and Asn524 while interacting with Trp516, Phe649, His674, Phe525, Leu678, and Leu 650 through pi-alkyl bonds and with Trp481 via pi–pi T-shaped bonds. Additionally, it established halogen bond interactions with Asp282, Ala555, Asp518, and His674 (Figures 3 and 4). These findings provide insights into the specific structural interactions between the synthesized compounds and the active site residues of the enzyme, which could contribute to their inhibitory effects on α-amylase and α-glucosidase. Importantly, the amino acids responsible for these interactions during the molecular docking process align with interaction sites previously reported in the literature, further validating the potential efficacy of these compounds against α-glucosidase and α-amylase.^69,70^

### 3.4 MD Simulation Analysis

Molecular dynamics (MD) simulation is a powerful computational method widely used to study the dynamic behavior of molecular systems over time. This technique enables detailed assessments of protein–ligand complexes, evaluating their stability and binding affinity at an atomic level.^71^ This study conducted MD simulations using the Desmond software package to investigate the binding affinity and stability of the most active compound **3** and the reference compound acarbose when complexed with α-glucosidase and α-amylase. The simulations spanned a 100 ns period, providing comprehensive insights into these complexes’ structural and dynamic characteristics.

**Figure 5:**
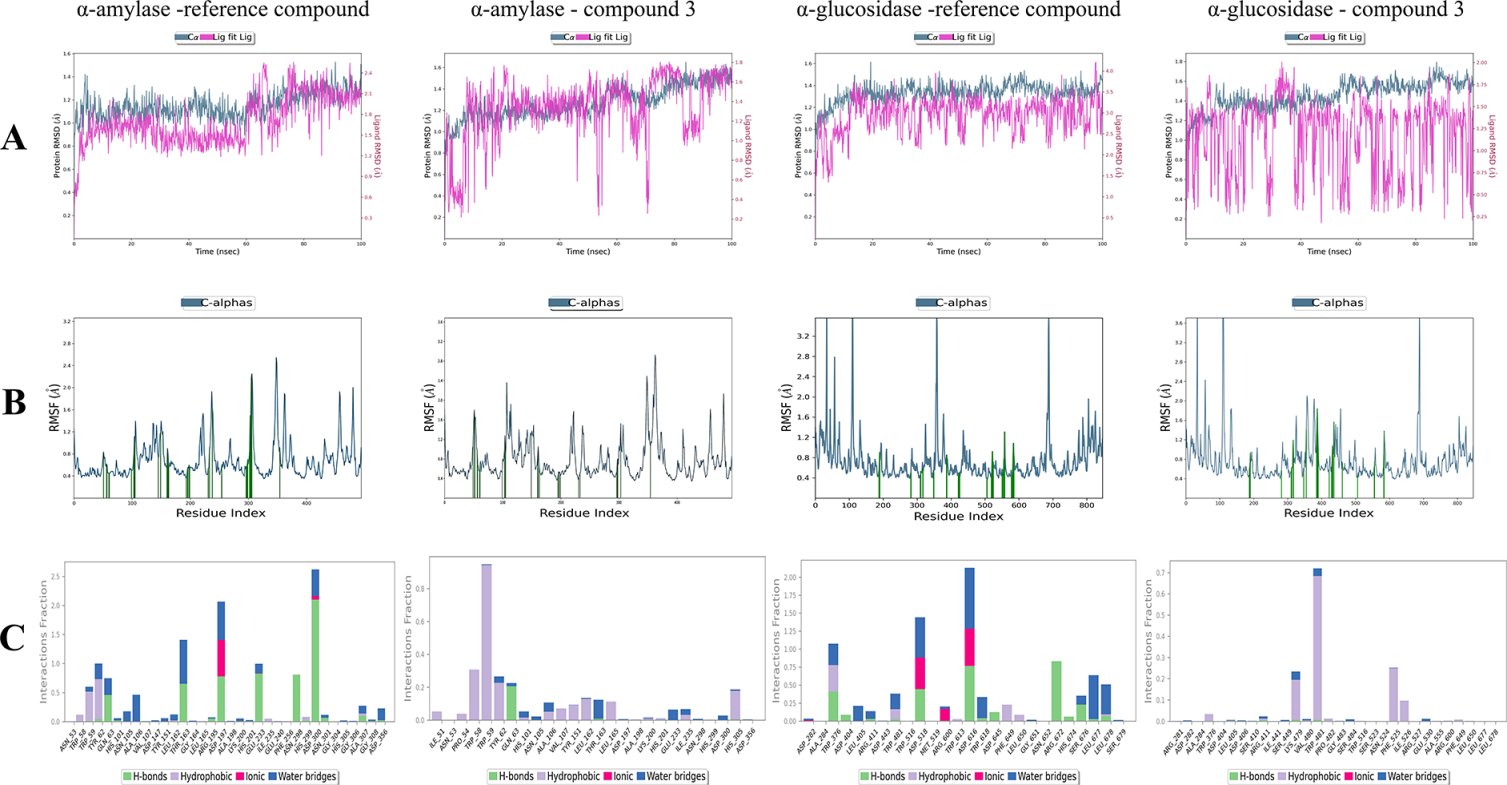
(A) RMSD calculation, represented by the protein’s blue line and the ligand’s red line, was conducted for each complex during the 100 ns MD simulation. (B) RMSF assessment was performed for each complex following a 100 ns MD simulation. (C) The four main factors in protein–ligand interaction are hydrogen bonds, hydrophobic contacts, ionic interactions, and water bridges. Stacked bar graphs visualize these interactions throughout the trajectory, indicating the duration as a percentage of simulation time.

RMSD analysis was performed to evaluate the structural stability of the protein–ligand complexes, offering a measure of conformational deviations from the initial structure throughout the simulation. The RMSD values for the Cα atoms of the α-amylase protein, in complex with compound **3** and the reference compound, ranged from 0.2 to 1.6 Å, reflecting minimal structural deviations throughout the simulation. Similarly, for the α-glucosidase protein, the RMSD values range between 0.2 and 1.8 Å for both compounds, indicating stable conformations during the simulation (Figure 5A). Additionally, root-mean-square fluctuation (RMSF) analysis was performed to evaluate the flexibility of individual protein residues in the presence of the tested compounds. Residues interacting with compound **3** and the reference compound exhibited RMSF values ranging from 0.4 to 2.8 Å for the α-amylase protein and from 0.4 to 3.2 Å for the α-glucosidase protein, as illustrated in Figure 5B.

Noncovalent interactions between the proteins and ligands were monitored throughout the simulation to elucidate binding affinities and the stability of the ligands within the active sites. Key catalytic residues in α-amylase, including Asp197, Glu233, and Asp300, known for their roles in enzymatic activity.^72^ maintained stable interactions with both compound **3** and the reference compound throughout the simulation (Figure 5C). Similarly, critical residues in α-glucosidase, such as Arg600, Asp616, Asp282, Met519, Ser528, Ser679, Trp618, Glu651, and Trp481,^73^ sustained noncovalent interactions with both compounds over the simulation period.

These results demonstrate that compound **3** and the reference compound exhibit stable binding within the catalytic domains of α-amylase and α-glucosidase. The consistency of these interactions, as supported by RMSD and RMSF analyses, underscores the compounds’ strong binding affinity and highlights their potential as effective inhibitors of these enzymes.

### 3.5 *In Silico* Studies

**Figure 6:**
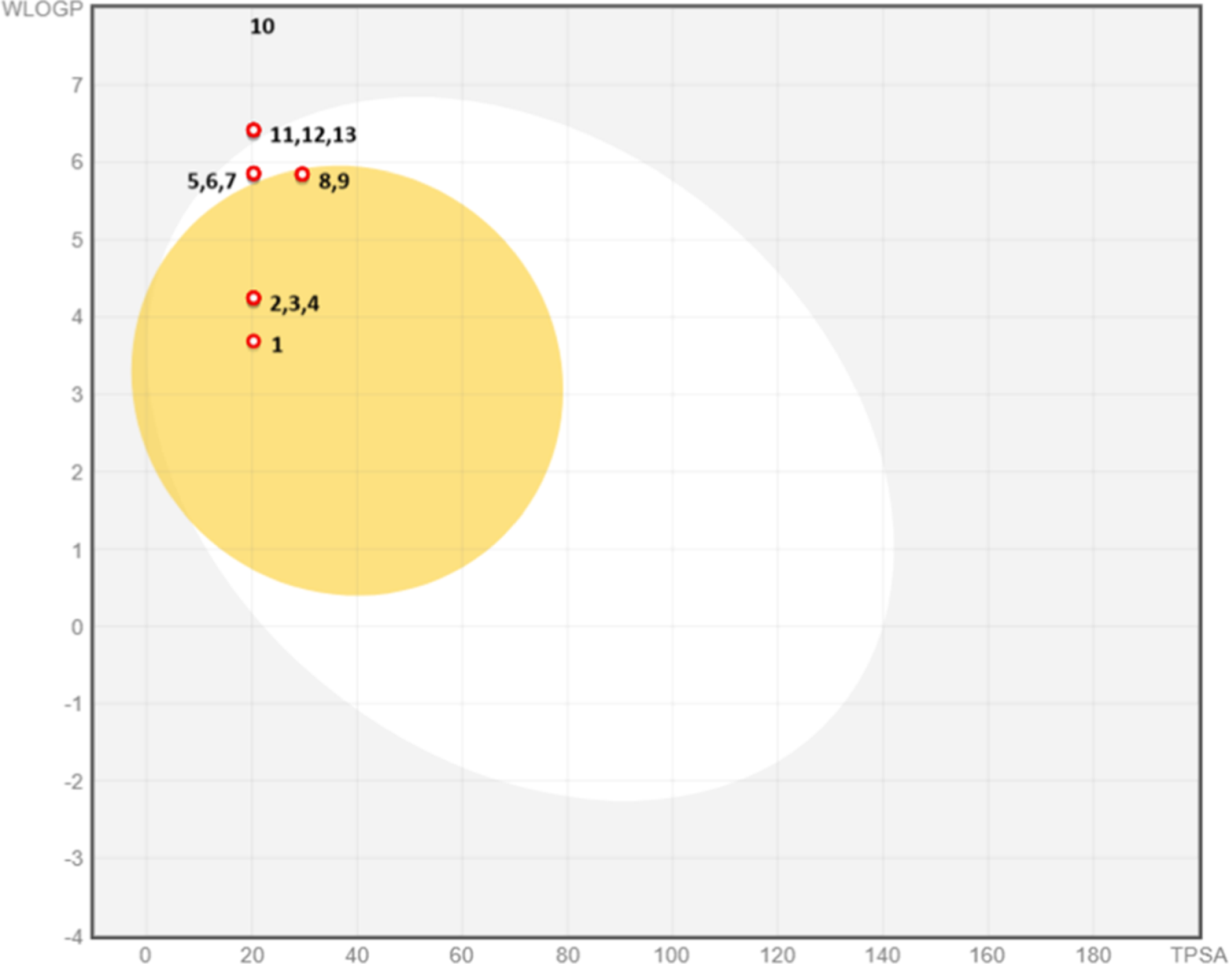
BOILED-Egg plot of chalcones (**1**–**13**).

One of the most important factors for a molecule to be a drug candidate is its suitability, in terms of pharmacokinetics. The physicochemical properties and ADME parameter values of the synthesized compounds **1**–**13** were examined. It was seen that their molecular weights were between 277.36 and 413–36 g/mol (150 < MA < 500); the number of hydrogen donors was 0 in all molecules (*n*–OHNH < 5); the number of hydrogen bond acceptors was 1–7 (n-ON < 10); topological polar surface area was 20.31 Å^2^ (TPSA < 70 Å^2^) in all compounds; MLOGP value was in the range of 3.30–4.98 (MLOGP ≤ 4.15); rotatable bonds were in the range of 4–6 (rotatable bonds <9) and were in the ideal range. Gastrointestinal absorption was determined to be high in compounds **1**–**9** and low in compounds **10**–**13**. While compounds **1**–**4**, **8**, and **9** cross the blood–brain barrier and will damage the central nervous system, causing things like depression and nervous disorders; compounds **5**, **6**, **7**, and **10**–**13** cannot cross the blood–brain barrier and will not harm the central nervous system. According to the BOILED-Egg plot, Compounds **5**, **6**, and **7** were in the white region, representing human intestinal absorption. Compounds **1**, **2**, **3**, **4**, **8**, and **9** were in the yellow region indicating strong intestinal absorption, and compounds **10**, **11**, **12**, and **13** were in the gray region indicating poor intestinal absorption (Figure 6, Table 3).^74,75^

**Table 3:** Predicted Physicochemical, Pharmacokinetics, and Drug Likeness Properties for the Chalcones (**1–13**)^a^

comp.	*M*_W_ (g/mol)	HBA (≤10)	HBD (≤5)	NRB (≤140A^2^)	TPSAÅ^2^	iLog P	GI absorption	BBB permeant	lipinski	synthetic accessibility
**1**	277.36	1	0	4	20.31	3.11	high	yes	0	2.44
**2**	295.35	2	0	4	20.31	3.19	high	yes	0	2.56
**3**	295.35	2	0	4	20.31	3.27	high	yes	0	2.53
**4**	295.35	2	0	4	20.31	3.21	high	yes	0	2.51
**5**	345.36	4	0	5	20.31	3.25	high	yes	1	2.84
**6**	345.36	4	0	5	20.31	3.44	high	yes	1	2.70
**7**	345.36	4	0	5	20.31	3.43	high	no	1	2.69
**8**	361.36	5	0	6	29.54	3.58	high	yes	0	2.72
**9**	361.36	5	0	6	29.54	3.53	high	yes	0	2.63
**10**	413.36	7	0	6	20.31	3.58	low	no	1	2.87
**11**	363.35	5	0	5	20.31	3.51	low	no	1	2.81
**12**	363.35	5	0	5	20.31	3.47	low	no	1	2.83
**13**	363.35	5	0	5	20.31	3.37	low	no	1	2.78

a *M*_W_ = molecular weights; HBA = H-bond acceptors; HBD = H-bond donors; NRB = Num. Rotatable bonds; TPSA = topological polar surface area; GI absorption = Gatrointestiral absorption; BBB permeant = brain barrier.

## 4 Conclusions

People with diabetes have a markedly increased chance of developing numerous types of cancer according to epidemiologic studies. Although many risk factors exist between T2DM and cancer, the current understanding of the potential biological relationship between the two diseases remains limited. Interestingly, the results from current studies have revealed that some drugs used to treat hyperglycemia may also be effective in the treatment of cancer.^60,76^ The development of drug resistance against drugs that have been used in the treatment of cancer and diabetes for many years and the undesirable side effects of these agents make long-term use of these drugs difficult, despite their increasing efficacy. Therefore, it is very important to find new anticancer and antidiabetic agents with low toxicity and few short- and long-term adverse effects on healthy tissues.

From these perspectives, a series of chalcones containing a pyrrolidine ring and fluorine substituents at different positions were designed and synthesized in good yields. Furthermore, their antidiabetic activities (α-amylase and α-glucosidase inhibition activities) and anticancer activities (A549 cell line) were investigated in this research. According to the results of α-amylase inhibitory activity, all compounds except compound **5** showed higher activity than acarbose, while all compounds except compounds **2**, **5**, **8**, **11**, and **13** exhibited higher α-glucosidase inhibitory activity compared to acarbose. Furthermore, compounds **5** (IC_50_: 82.20 μM) and **8** (IC_50_: 59.96 μM) showed better cytotoxic activity than did cisplatin against A549 cells. The toxic effects of these compounds on healthy BEAS-2B cells were compared with cisplatin and it was revealed that they did not have a toxic effect. It was also found from the *in silico* molecular docking analysis that the binding interactions with α-amylase and α-glucosidase were consistent with the experimental data. The potent compounds identified in *in vitro* studies have a high affinity for these specific binding sites, supporting their potential as effective inhibitors in regulating the α-amylase and α-glucosidase enzyme activity.

## Supporting Information Available

^1^H NMR, ^13^C NMR, FT-IR and Mass spectrum of compounds **1**–**13** (PDF)
