# Supplementary material for: Discovery of Novel Pyrrolidine-Based Chalcones as Dual Inhibitors of α-Amylase and α-Glucosidase: Synthesis, Molecular Docking, ADMET Profiles, and Pharmacological Screening
Source: ACS Omega. 2025 Feb 26;10(9):9368–80. doi: 10.1021/acsomega.4c10095 (PMC11904715; doi:10.1021/acsomega.4c10095)
Supplement: Supplementary file 1 — ao4c10095_si_001.pdf [file ao4c10095_si_001.pdf]

SUPPORTING INFORMATION

Discovery of novel pyrrolidine-based chalcones as dual inhibitors of α-amylase and α-glucosidase: Synthesis, molecular docking, ADMET profiles and pharmacological screening

Bedriye Seda Kurşun Aktar[a*], Yusuf Sıcak[b,], Abdulkadir Bakırdöven[a], Gizem Tatar Yılmaz[c,d,e], Özlem Yılmaz[f], Ayşegül Karaküçük-İyidoğan[g], Demet Taşdemir[h,ı] , Ebru Sağlam[ı], Emine Elçin Oruç-Emre[g]

[a]Department of Hair Care and Beauty Services, Yeşilyurt Vocational School, Malatya Turgut Özal University, Malatya, Türkiye

[b]Department of Medicinal and Aromatic Plants, Köyceğiz Vocational School, Mugla Sitki Kocman University, Köyceğiz, Muğla, Türkiye

[c]Department of Biostatistics and Medical Informatics, Faculty of Medicine, Karadeniz Technical University, Trabzon, 61080 Türkiye

[d]Department of Bioinformatics, Institute of Health Sciences, Karadeniz Technical University, 61080, Trabzon, Türkiye

[e]Yılmaz Bilişim R&D Consulting Software Engineering and Services Trade Limited Company 61081, Trabzon, Türkiye

[f]Department of Chemistry, Faculty of Arts and Sciences, Tokat Gaziosmanpaşa University, Tokat, Türkiye

[g]Department of Chemistry, Faculty of Arts and Sciences, Gaziantep University, Gaziantep, Türkiye

[h]Department of Medical Biochemistry, Faculty of Medicine, Gaziantep University, Gaziantep, Türkiye

[ı]Respiratory Diseases and Respiratory Surgery Research and Practice Center, Gaziantep University, Gaziantep, Türkiye

Email: bseda.kursunaktar@ozal.edu.tr

Corresponding Author: Bedriye Seda Kurşun Aktar

Address: Malatya Turgut Özal University

Yesilyurt Vocational School

Ikizce District Ikizce Kümeevler No 100/4

44900 Yesilyurt Malatya

Supporting Information

Contents

FT-IR, ^{1}H NMR, ^{13}C NMR and MASS spectrum of the synthesized compounds……….S2

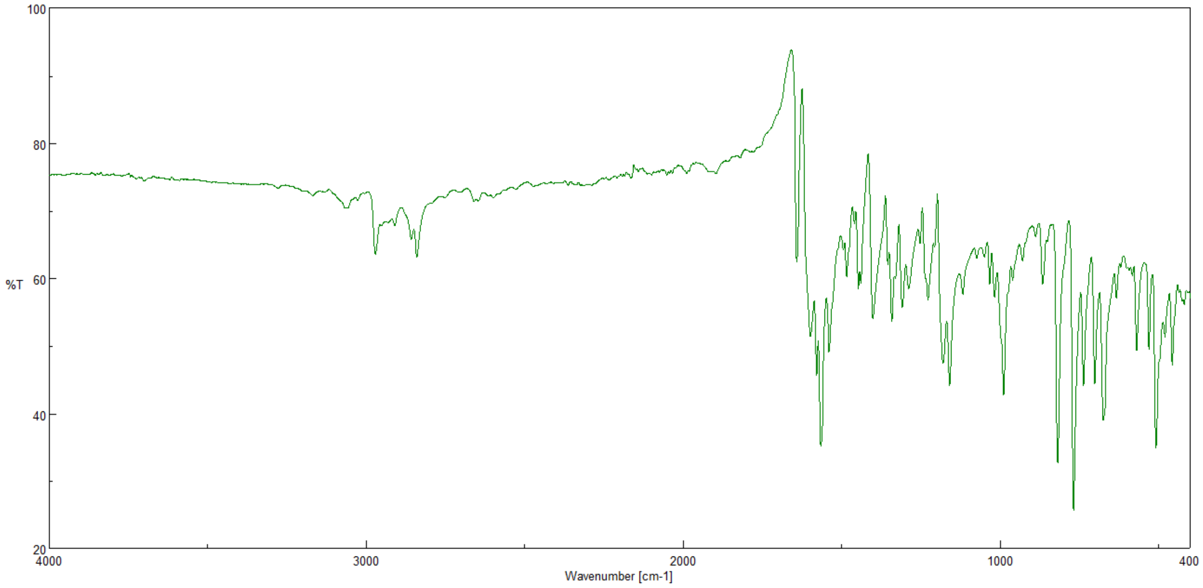

Figure S1. FTIR spectrum of compound **1**

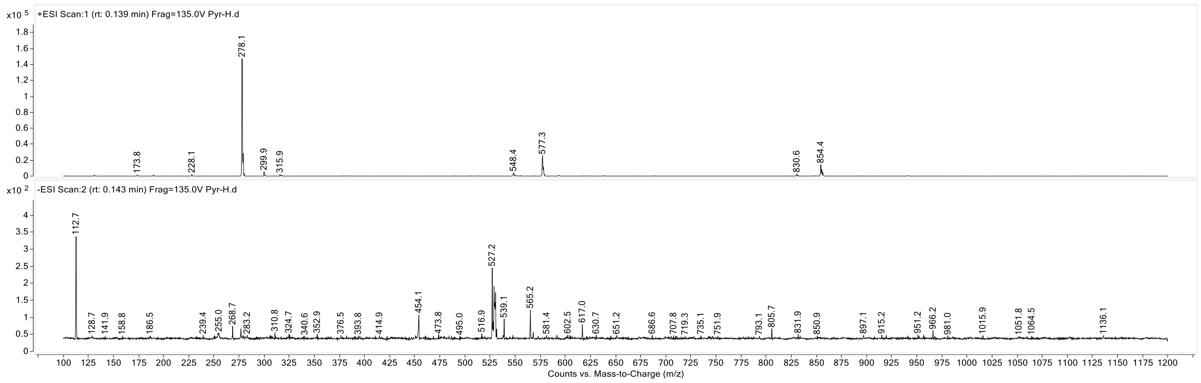

Figure S2. Mass spectrum of compound **1**

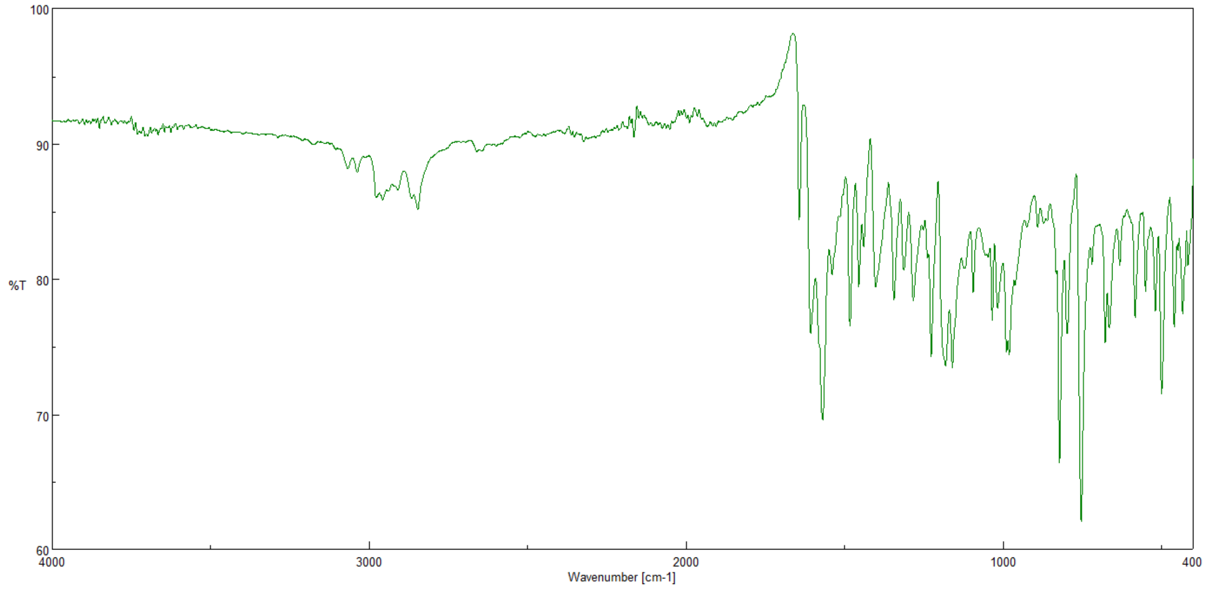

Figure S3. FTIR spectrum of compound **2**

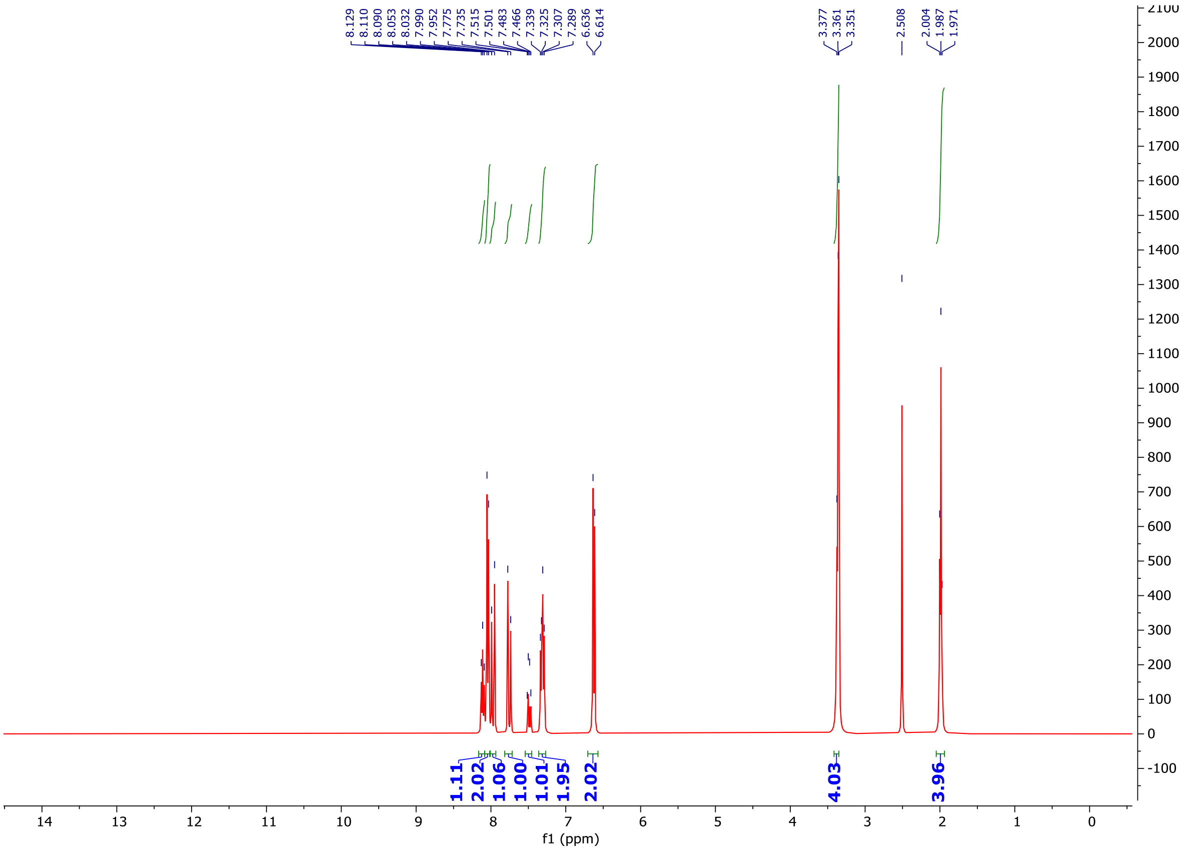

Figure S4. ^{1}H NMR spectrum of compound **2**

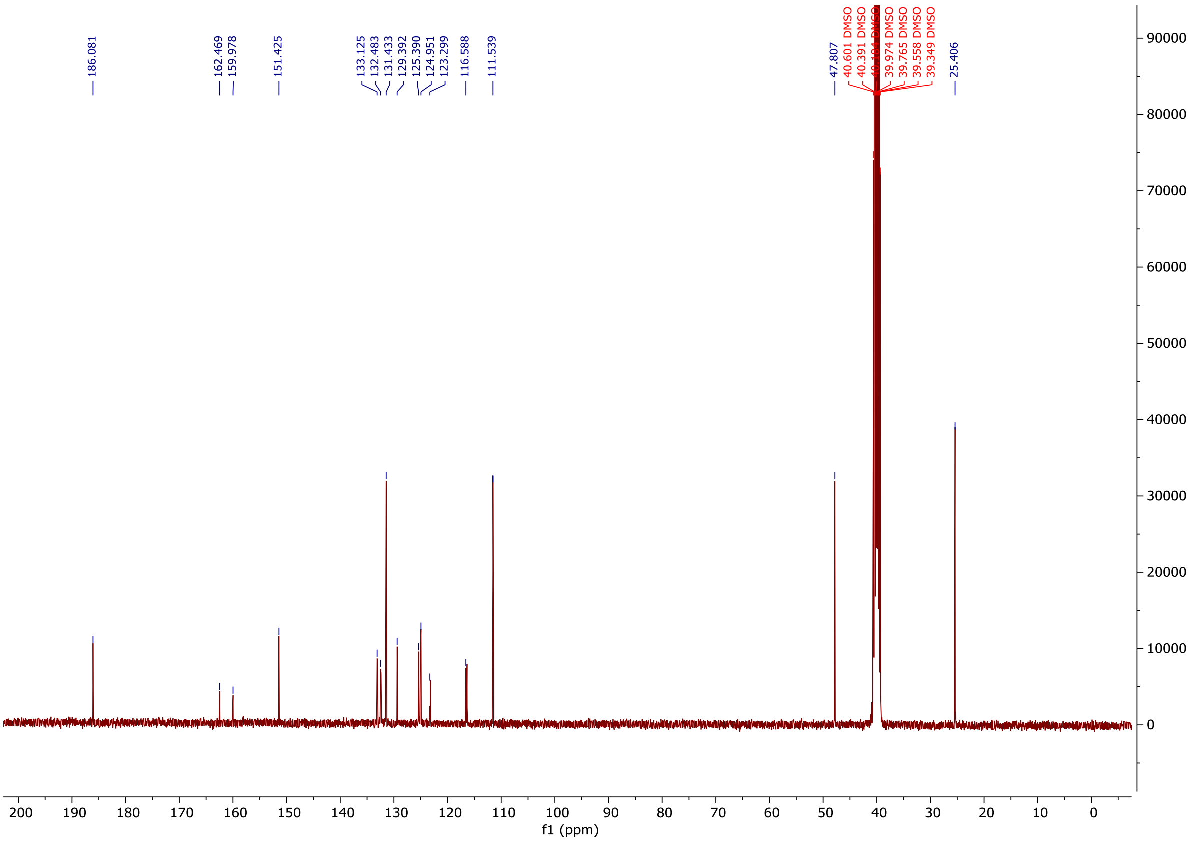

Figure S5. ^{13}C NMR spectrum of compound **2**

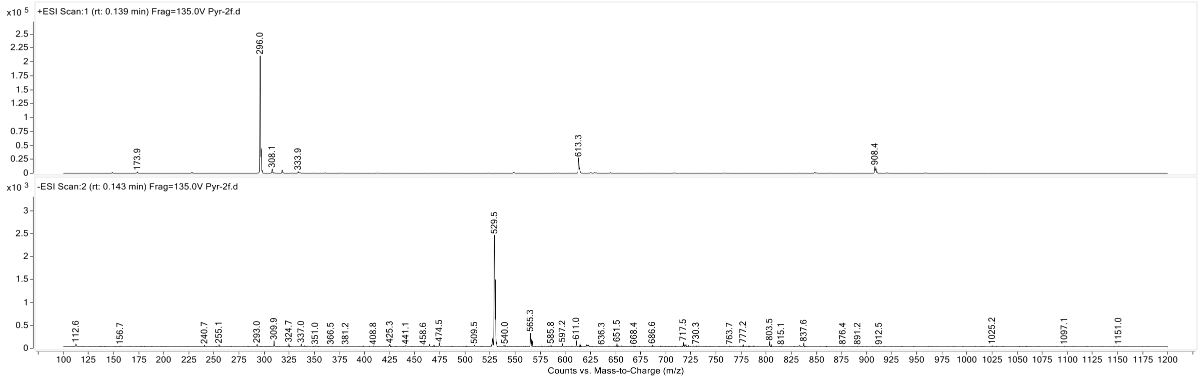

Figure S6. Mass spectrum of compound **2**

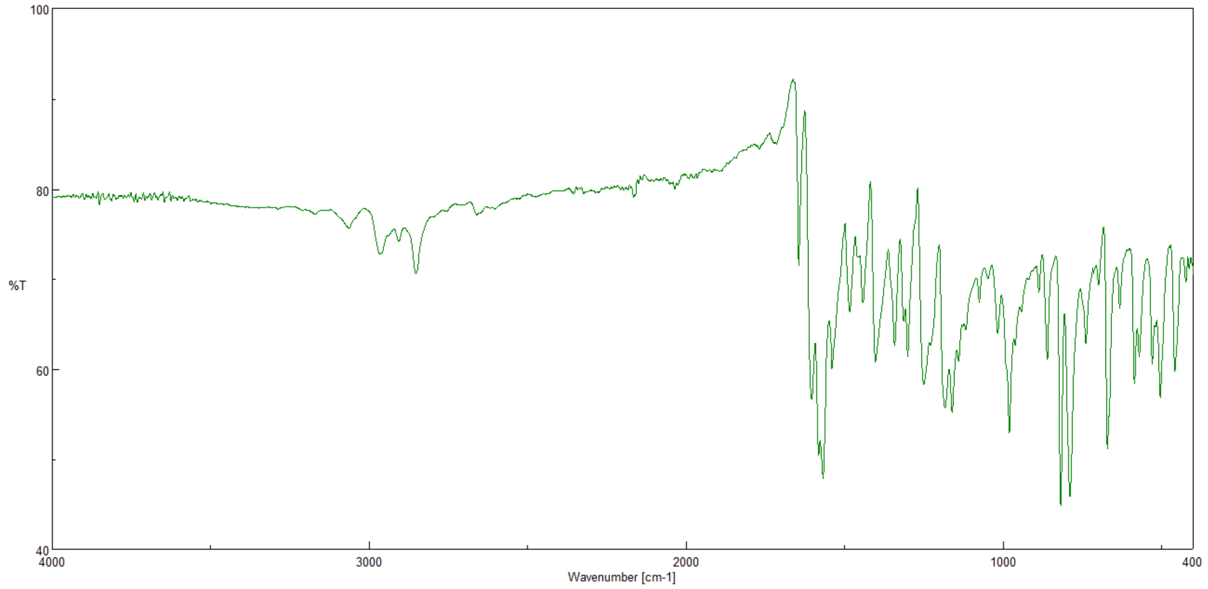

Figure S7. FTIR spectrum of compound **3**

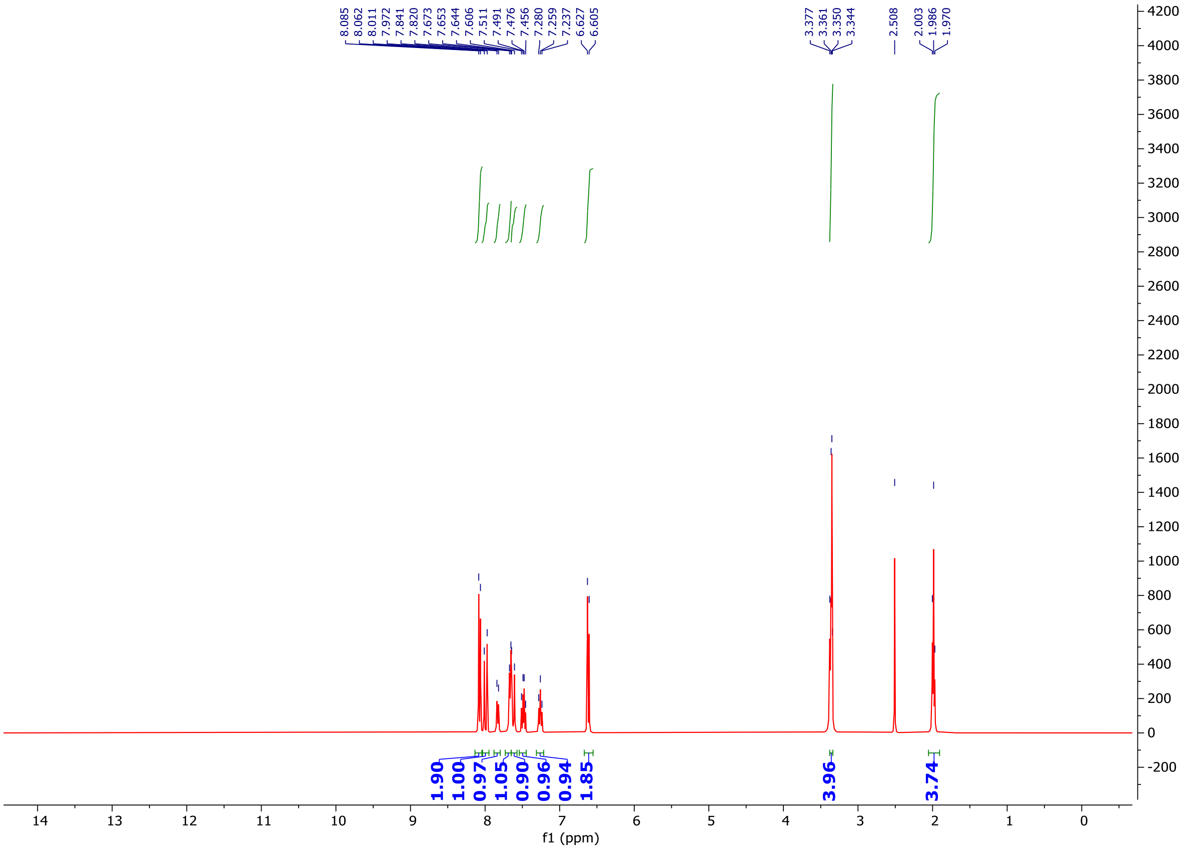

Figure S8. ^{1}H NMR spectrum of compound **3**

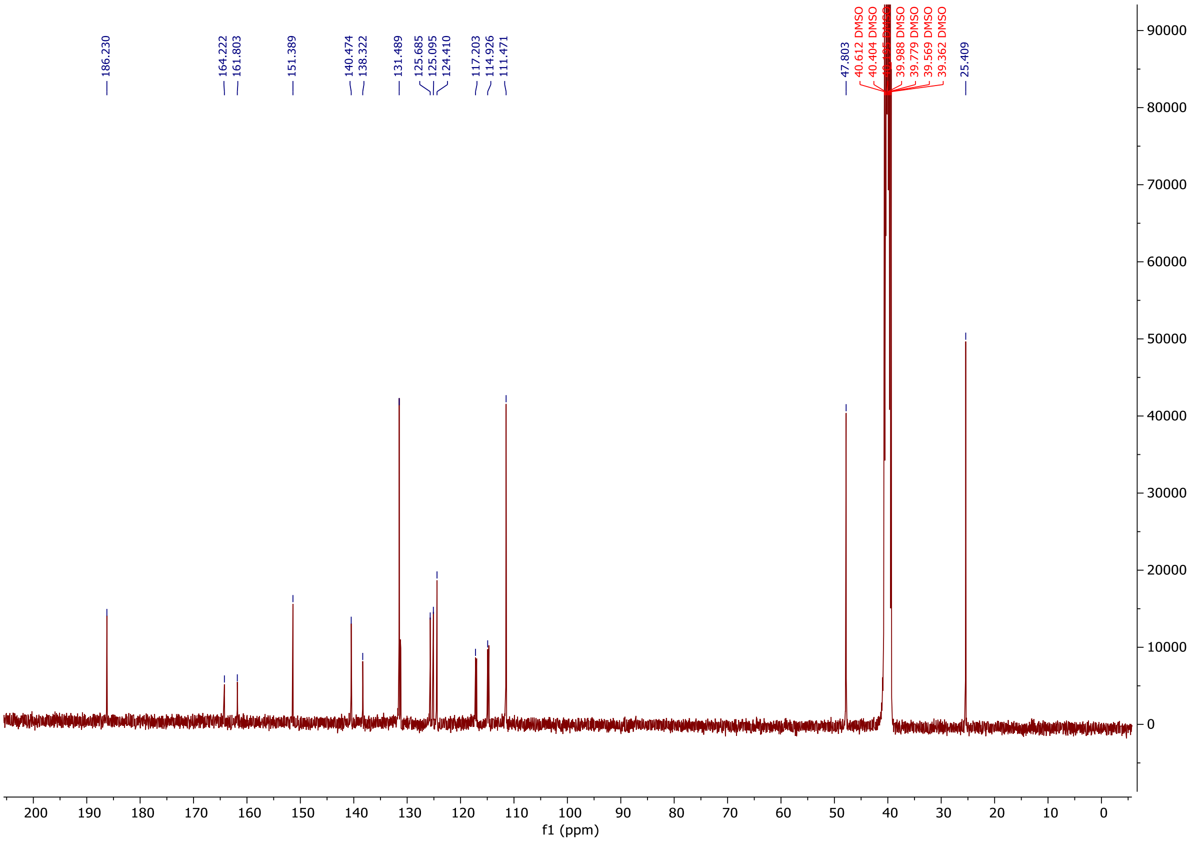

Figure S9. ^{13}C NMR spectrum of compound **3**

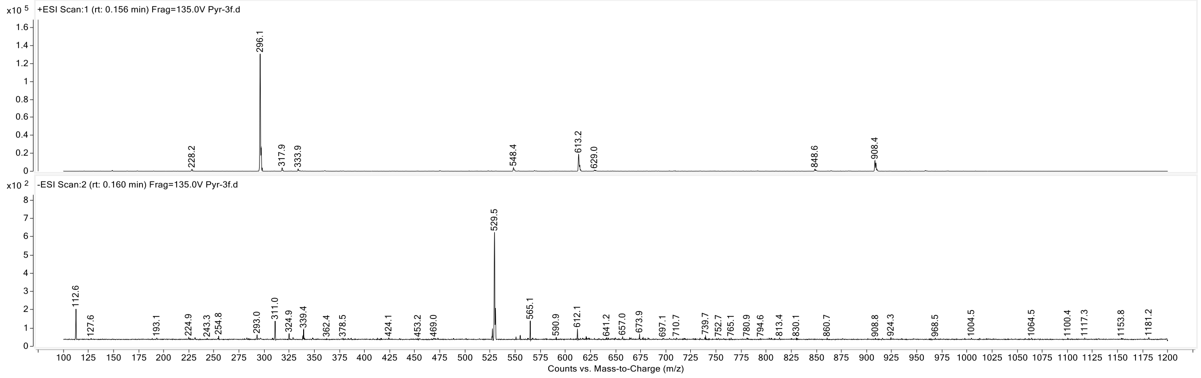

Figure S10. Mass spectrum of compound **3**

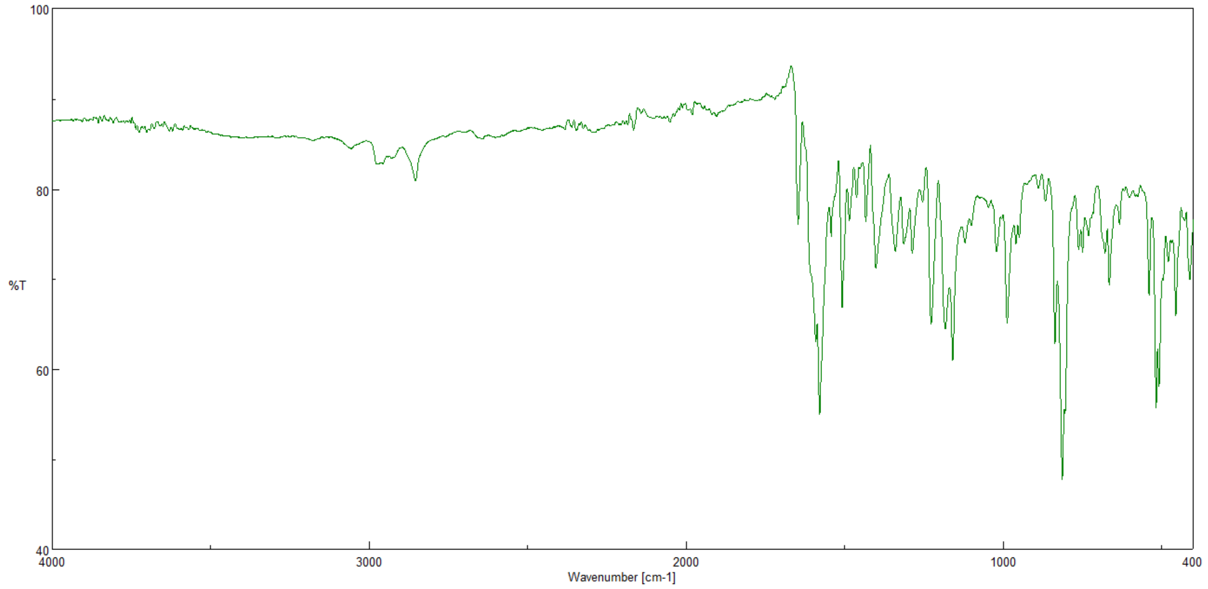

Figure S11. FTIR spectrum of compound **4**

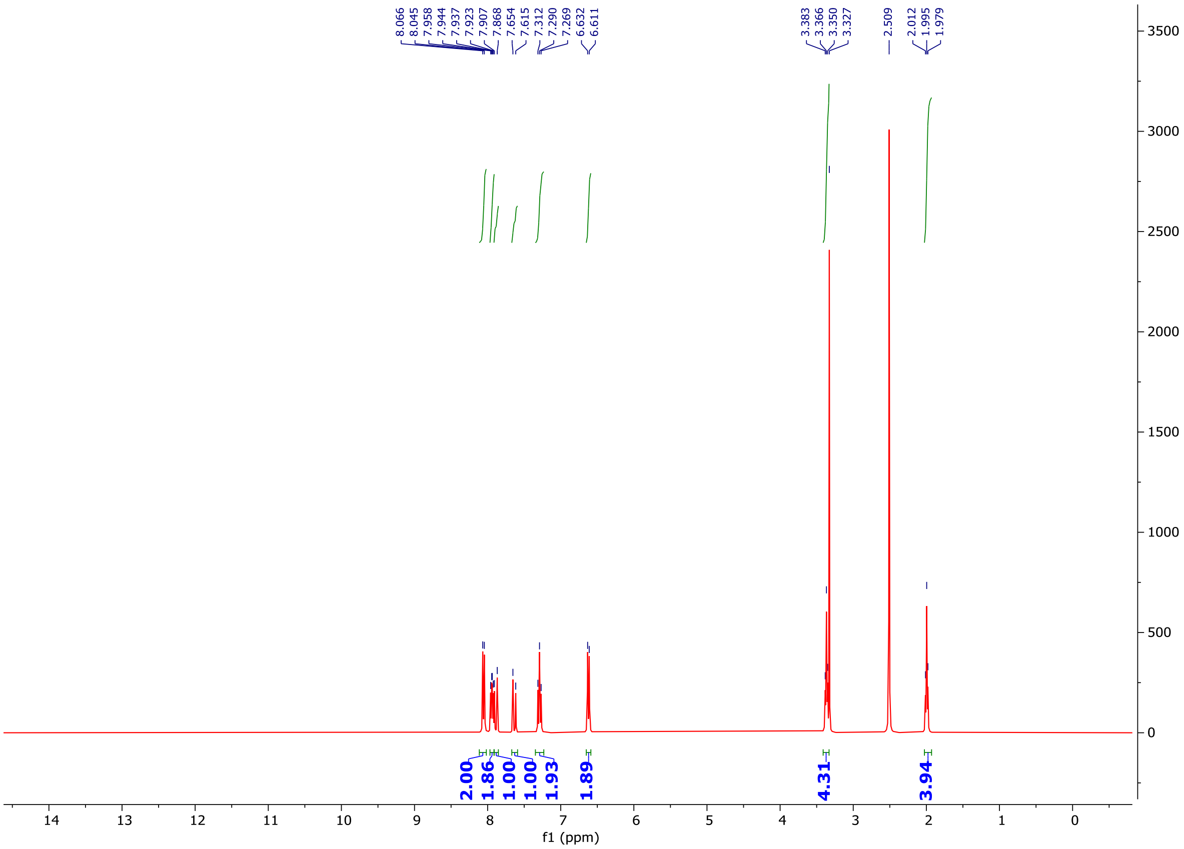

Figure S12. ^{1}H NMR spectrum of compound **4**

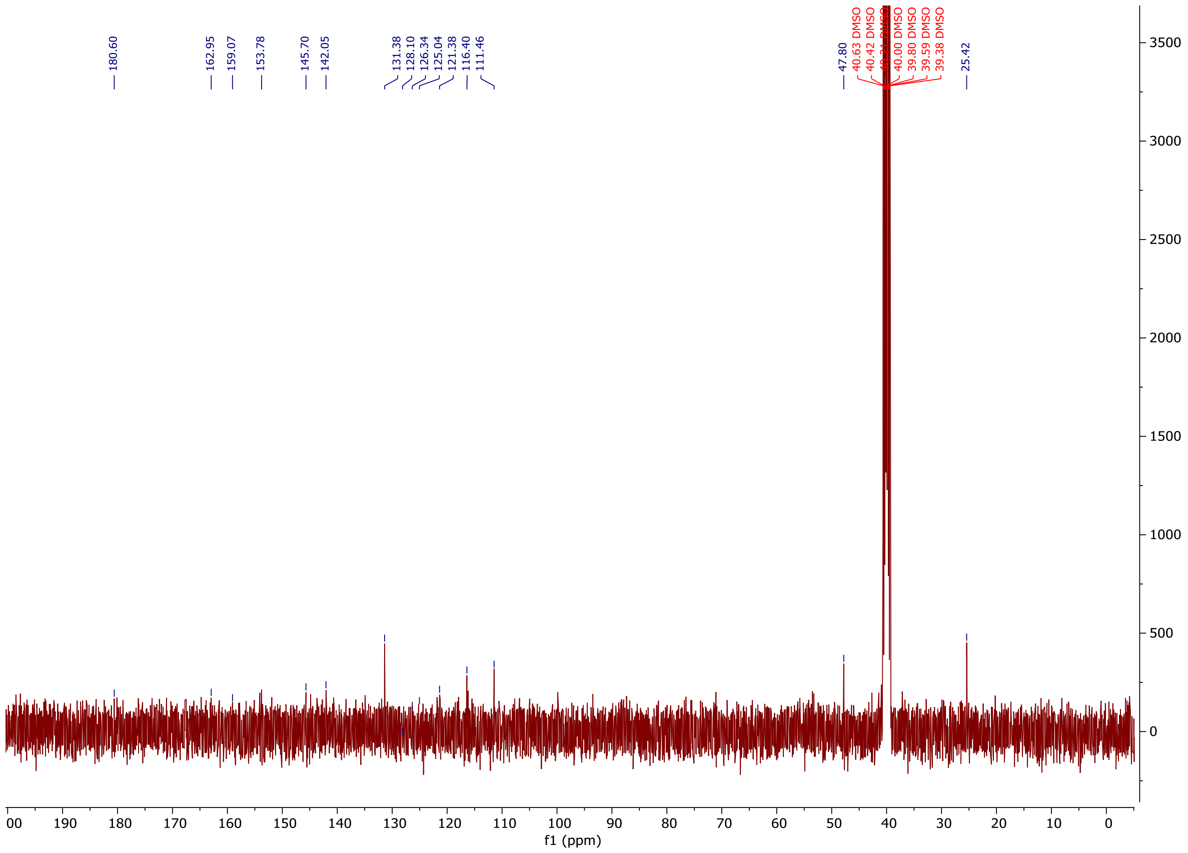

Figure S13. ^{13}C NMR spectrum of compound **4**

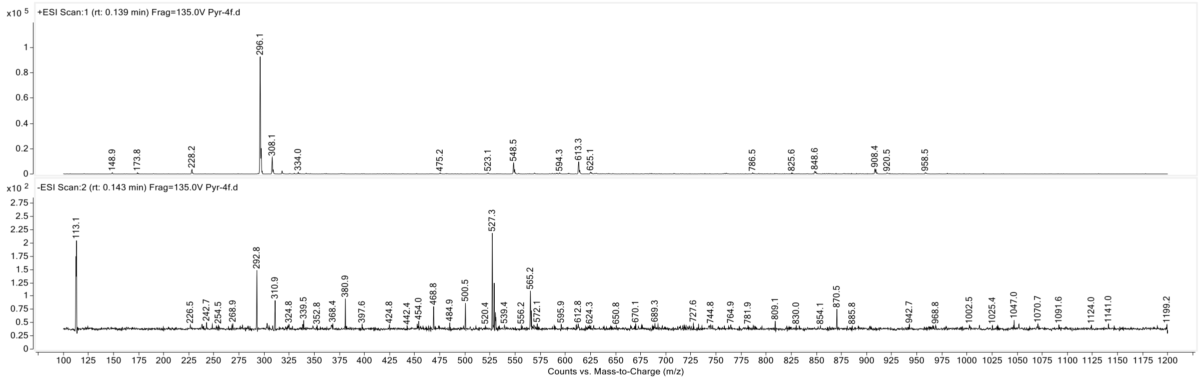

Figure S14. Mass spectrum of compound **4**

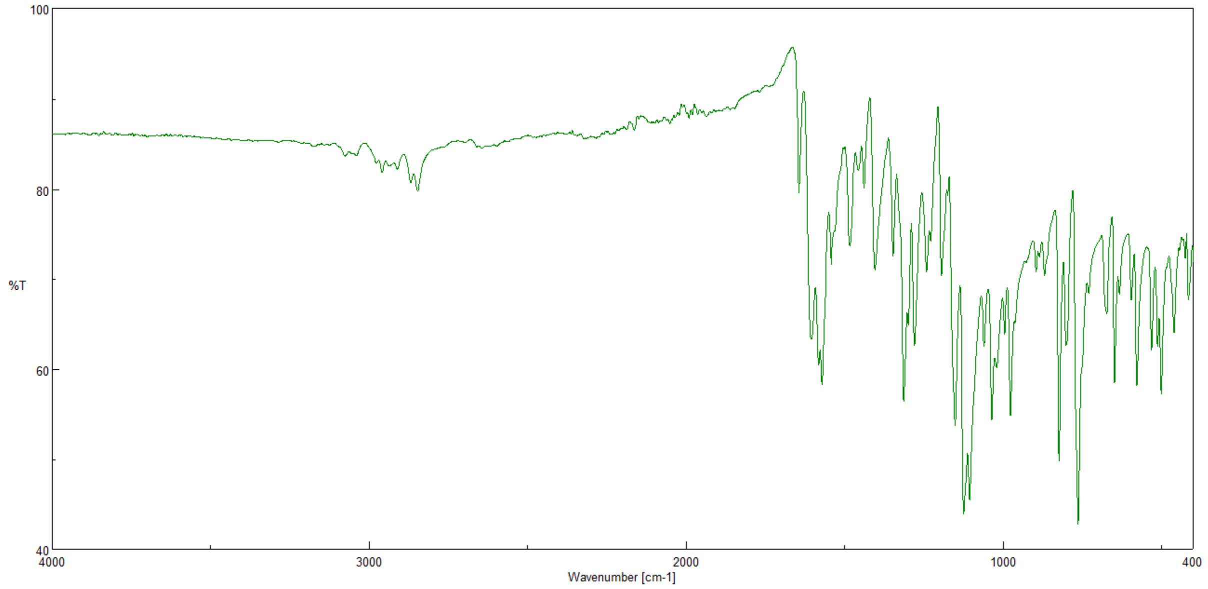

Figure S15. FTIR spectrum of compound **5**

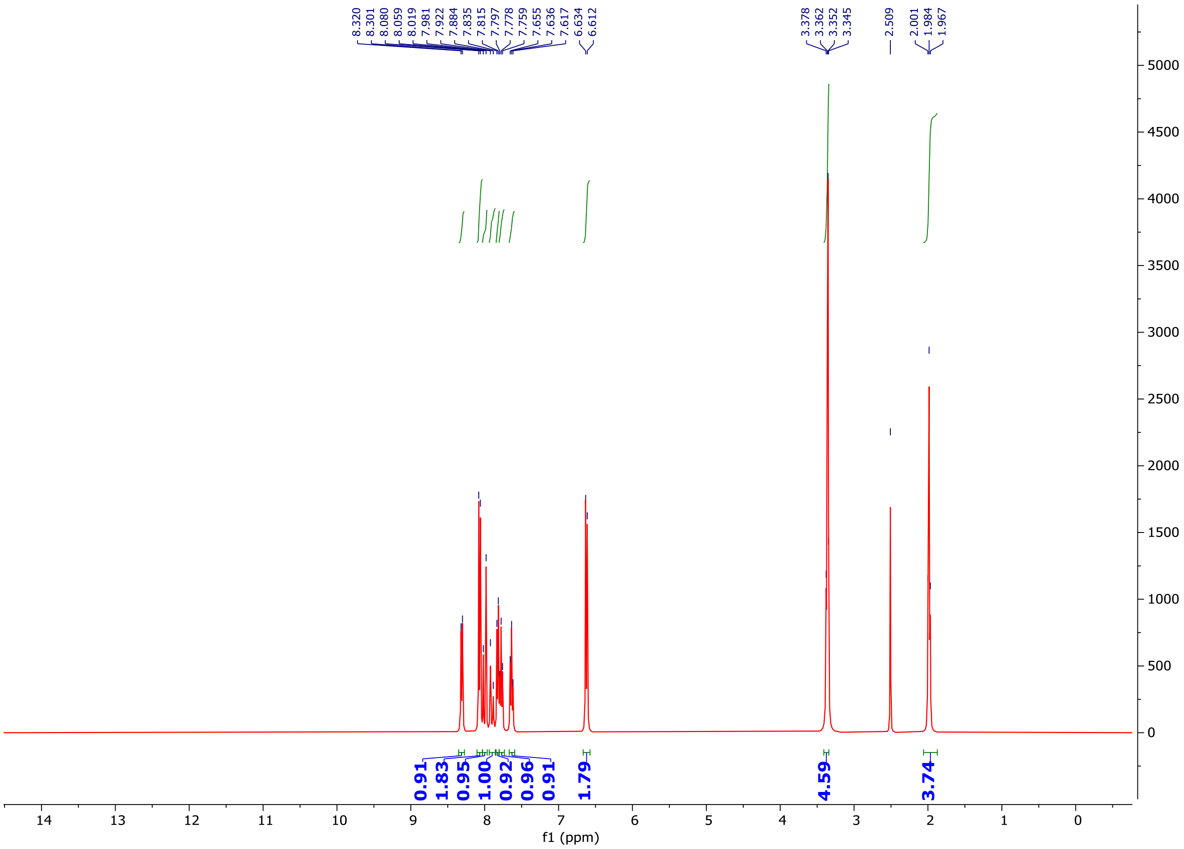

Figure S16. ^{1}H NMR spectrum of compound **5**

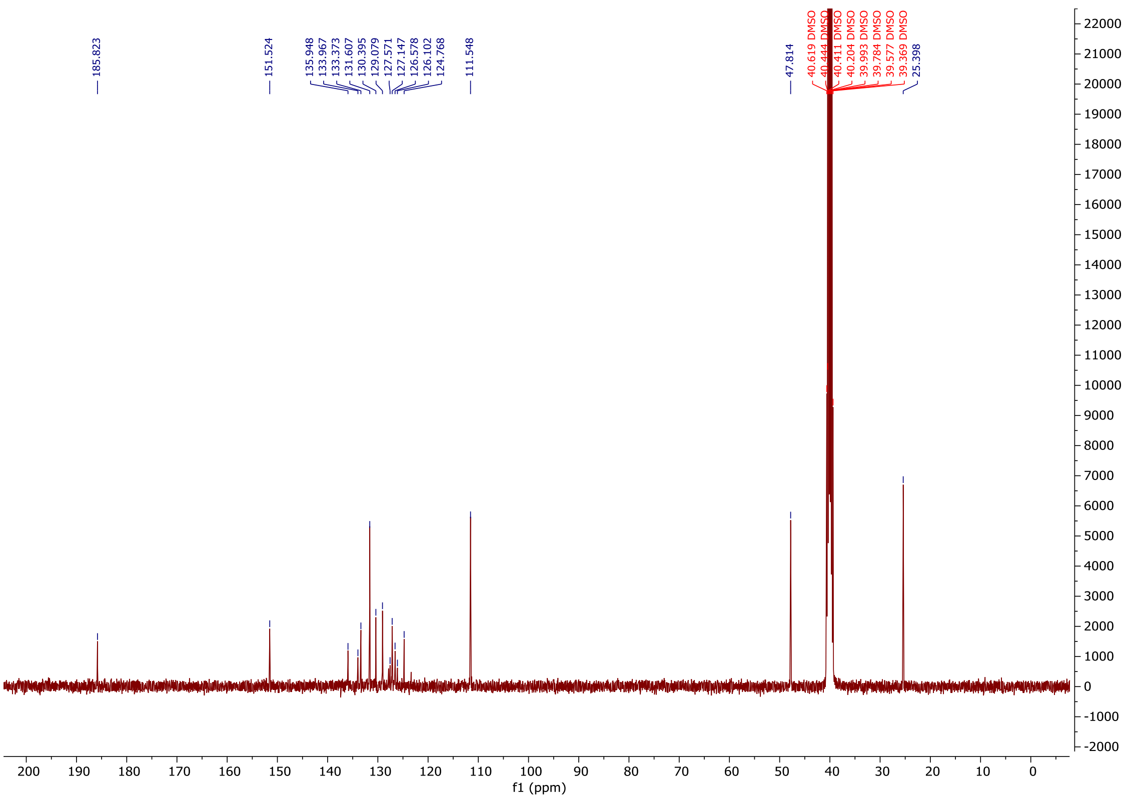

Figure S17. ^{13}C NMR spectrum of compound **5**

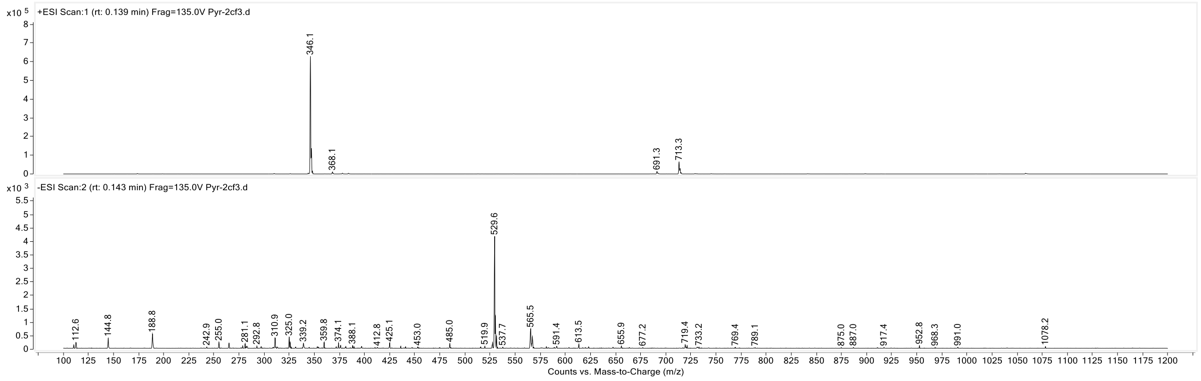

Figure S18. Mass spectrum of compound **5**

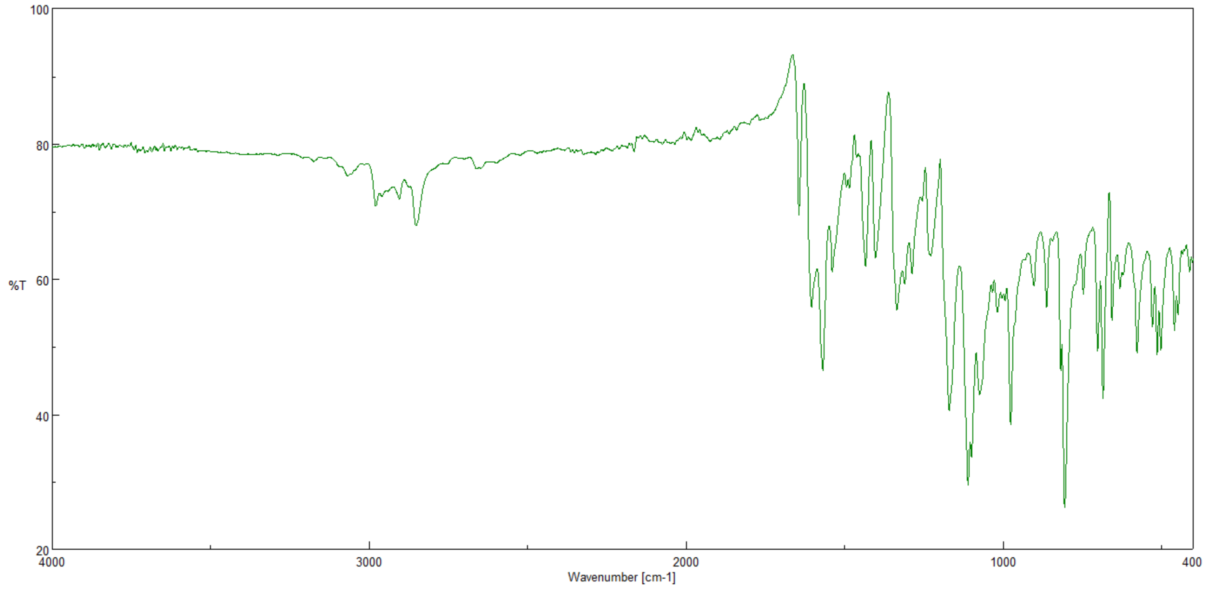

Figure S19. FTIR spectrum of compound **6**

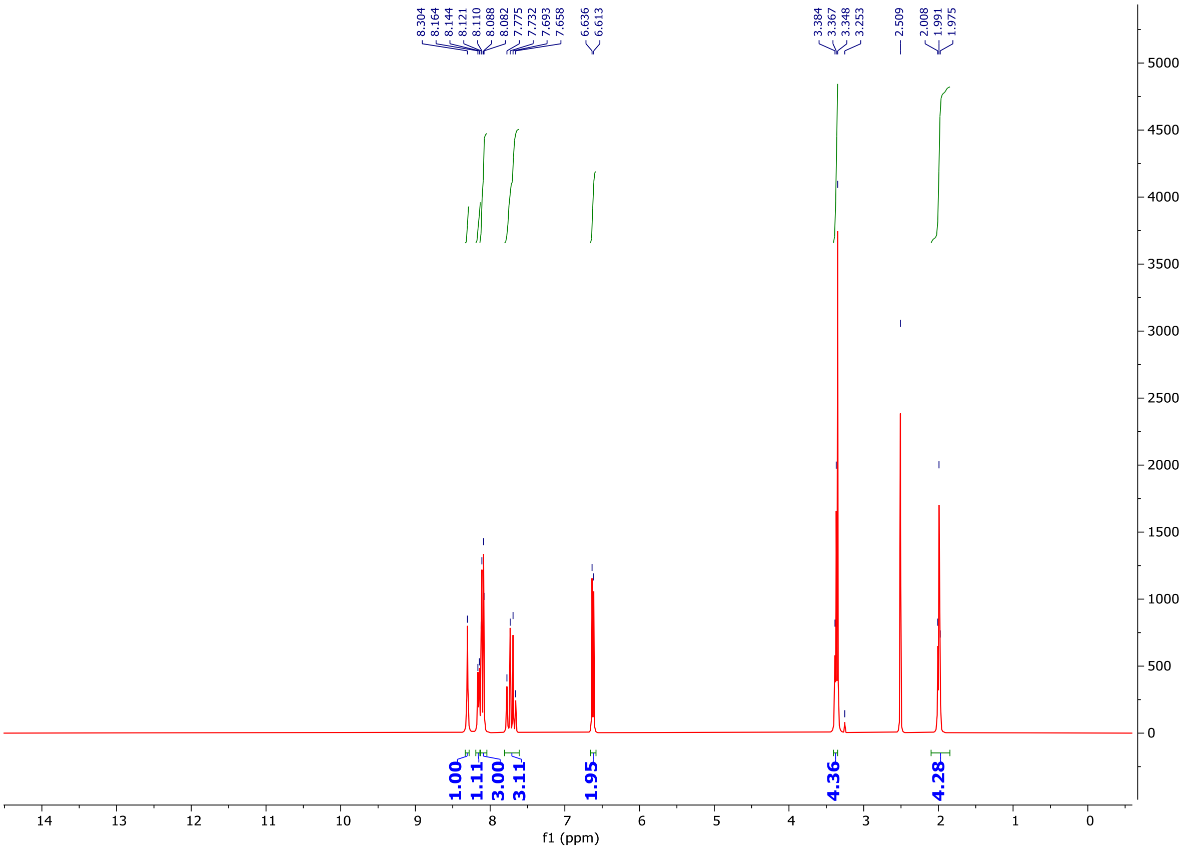

Figure S20. ^{1}H NMR spectrum of compound **6**

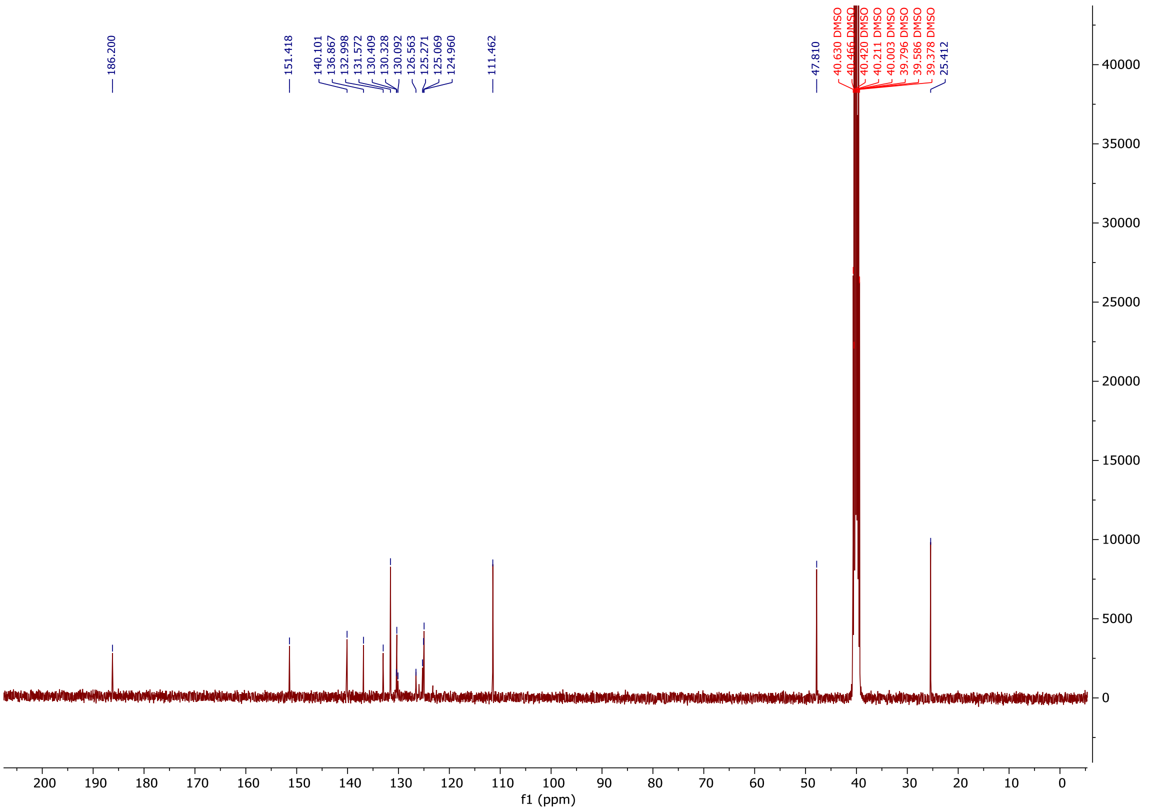

Figure S21. ^{13}C NMR spectrum of compound **6**

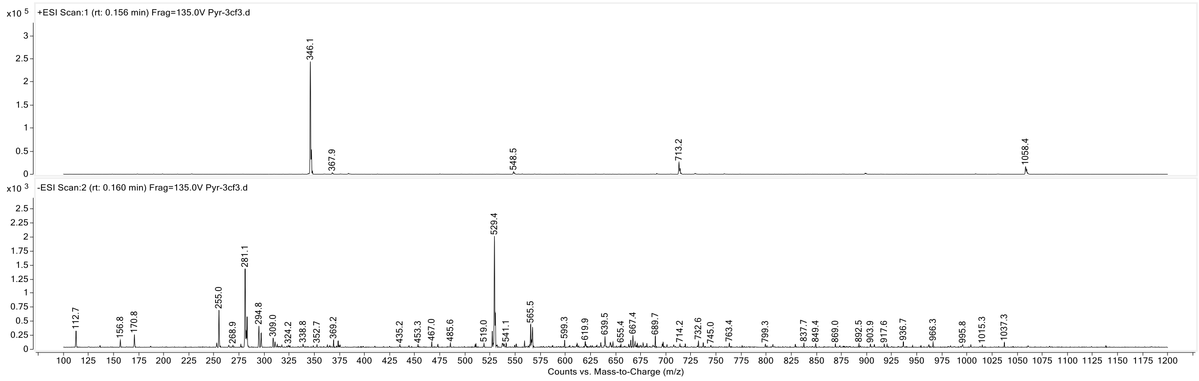

Figure 22. Mass spectrum of compound **6**

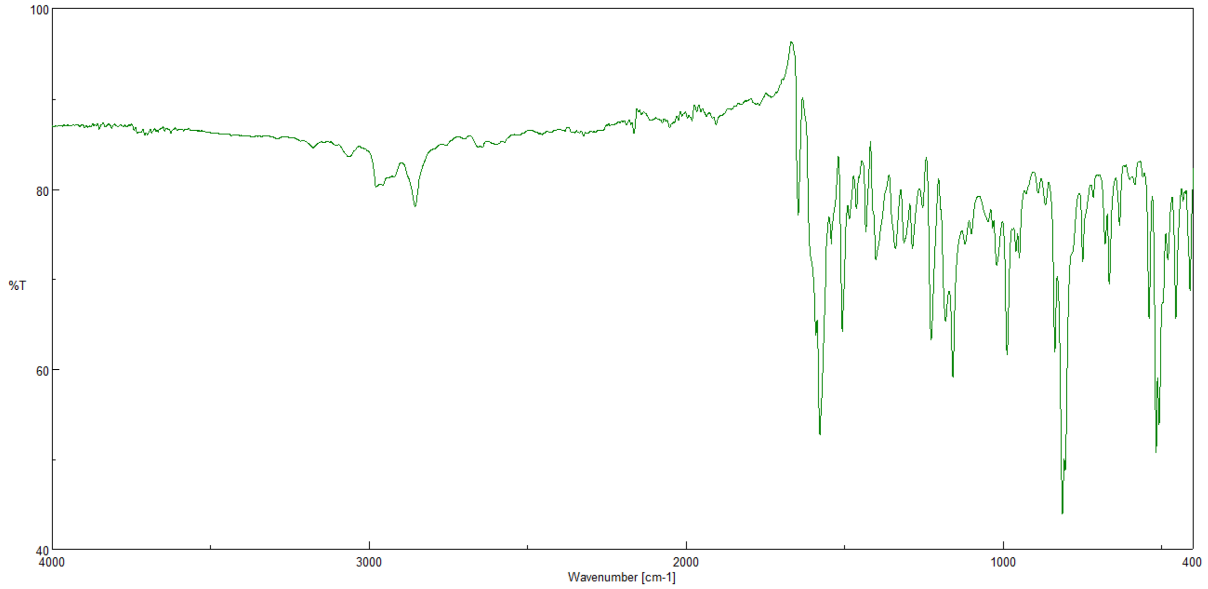

Figure S23. FTIR spectrum of compound **7**

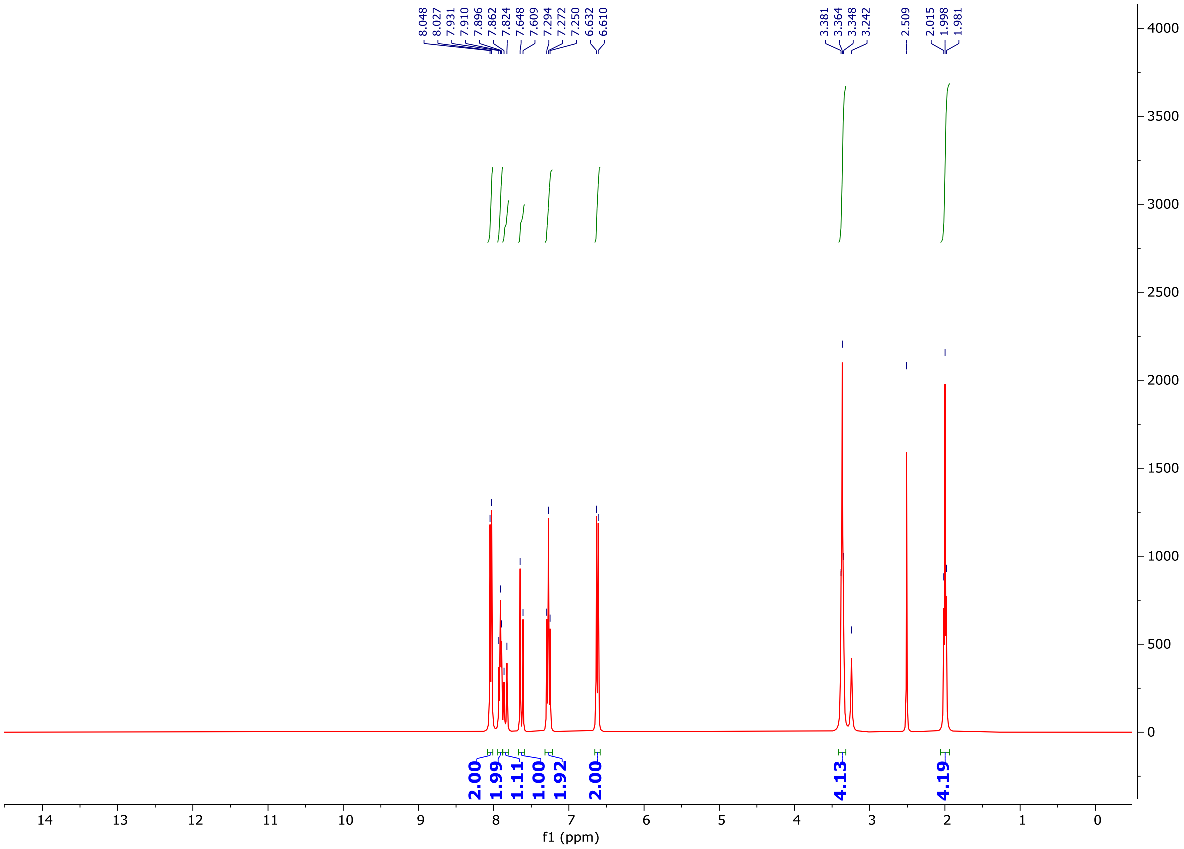

Figure S24. ^{1}H NMR spectrum of compound **7**

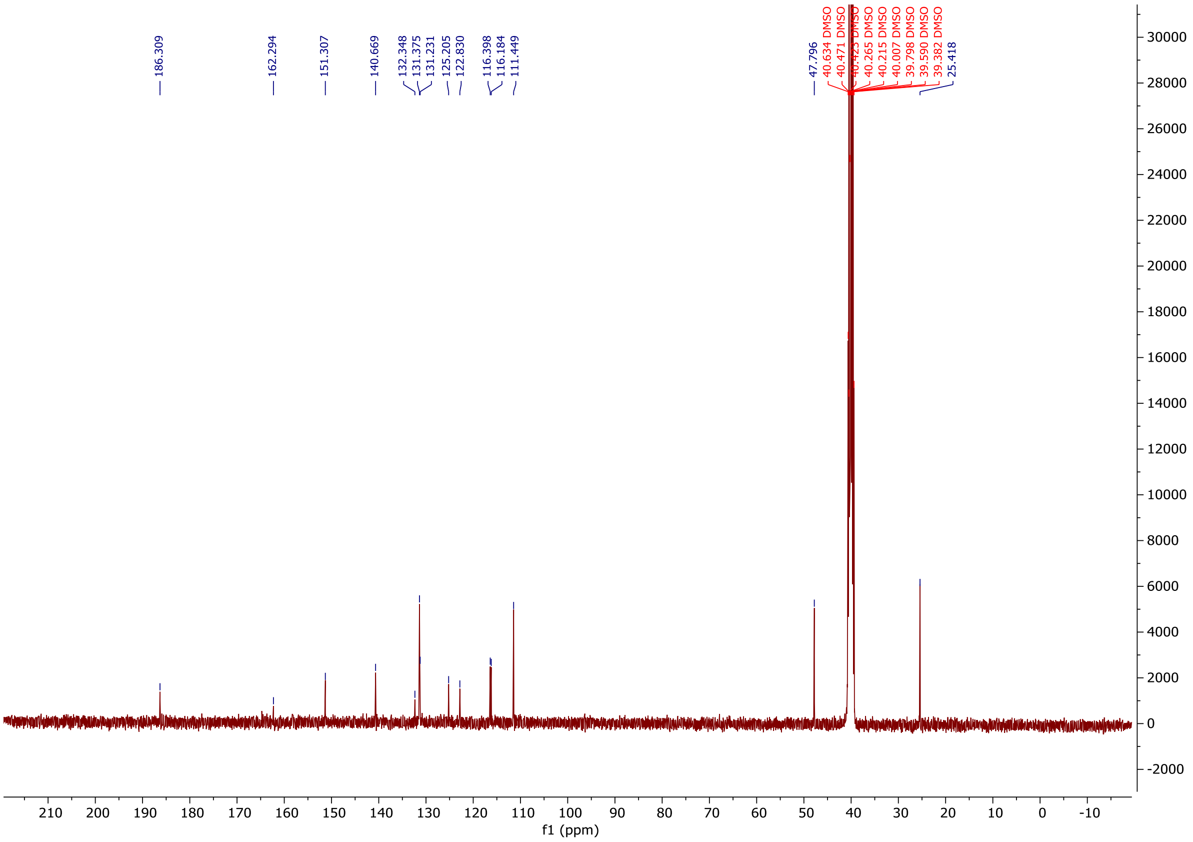

Figure S25. ^{13}C NMR spectrum of compound **7**

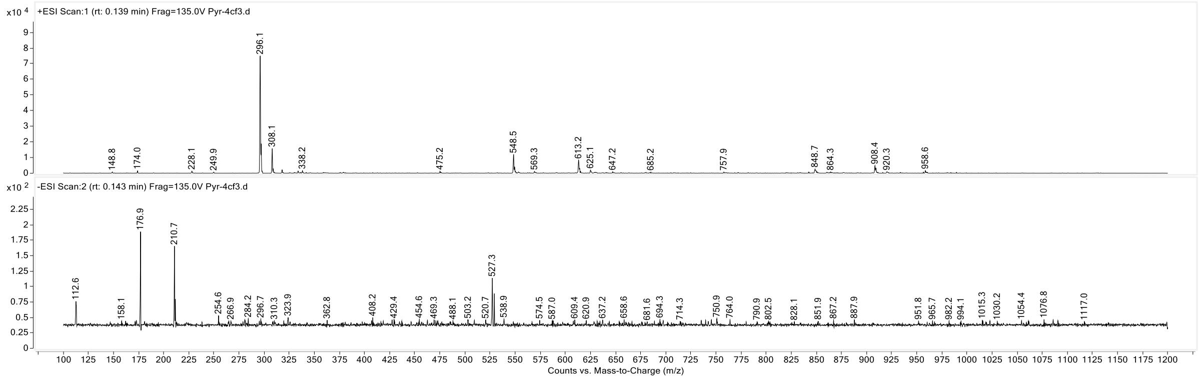

Figure S26. Mass spectrum of compound **7**

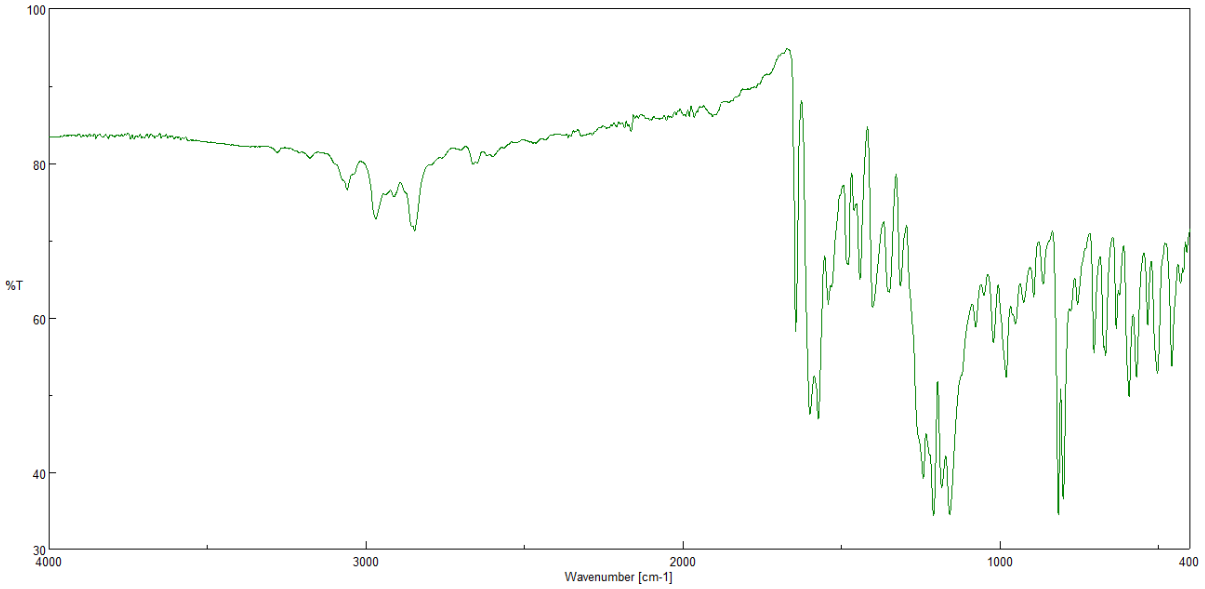

Figure S27. FTIR spectrum of compound **8**

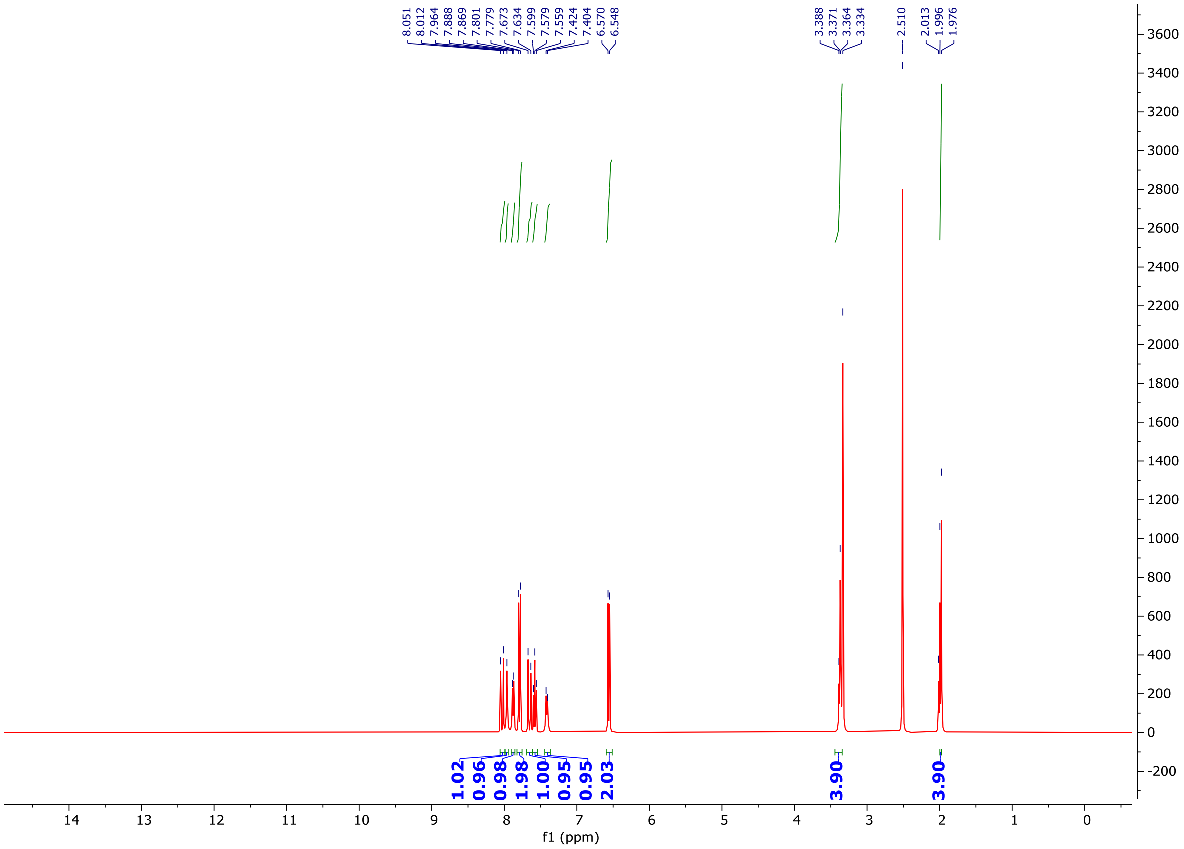

Figure S28. ^{1}H NMR spectrum of compound **8**

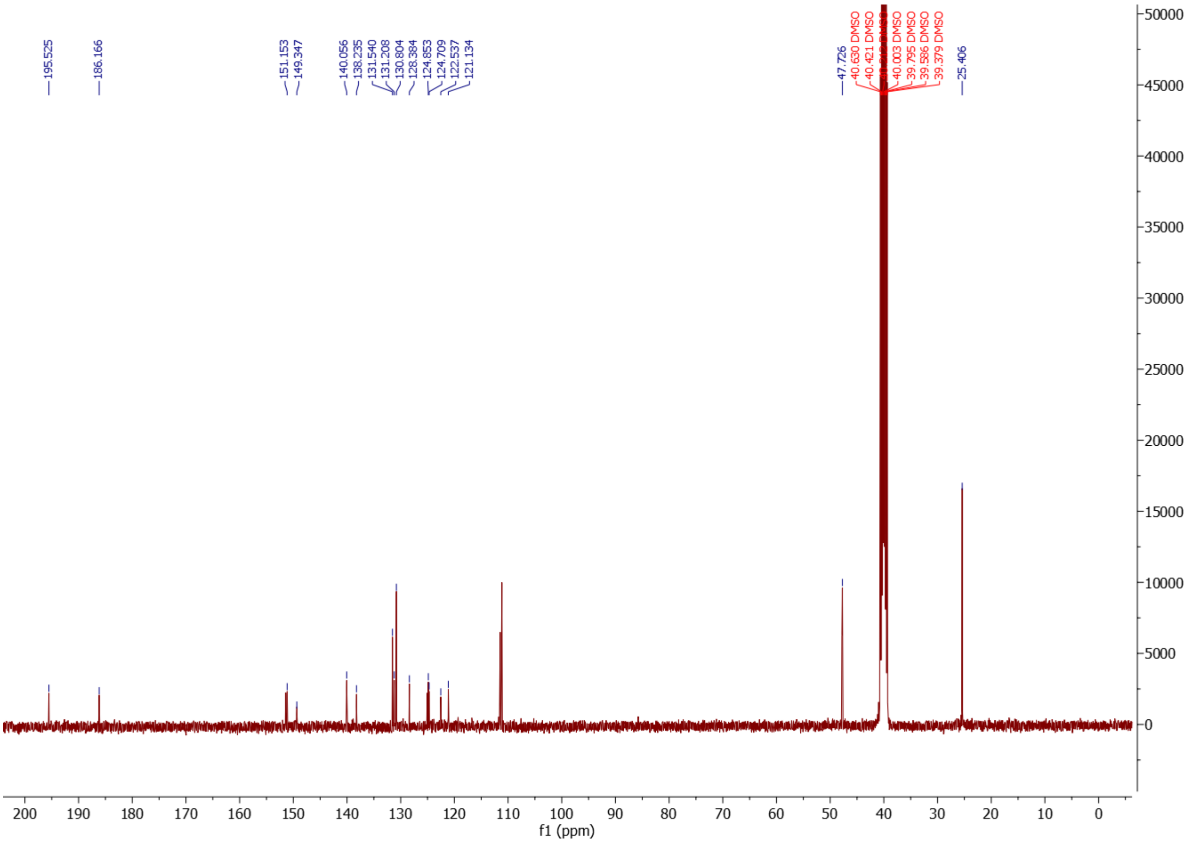

Figure S29. ^{13}C NMR spectrum of compound **8**

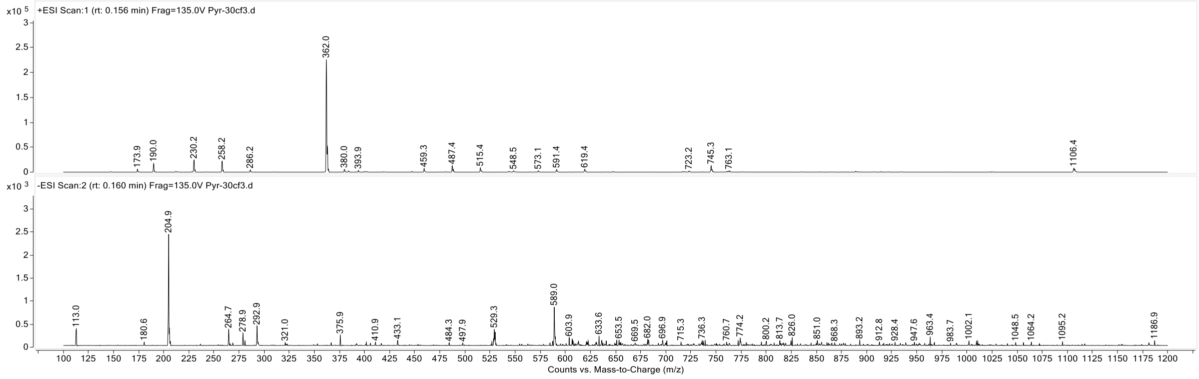

Figure S30. Mass spectrum of compound **8**

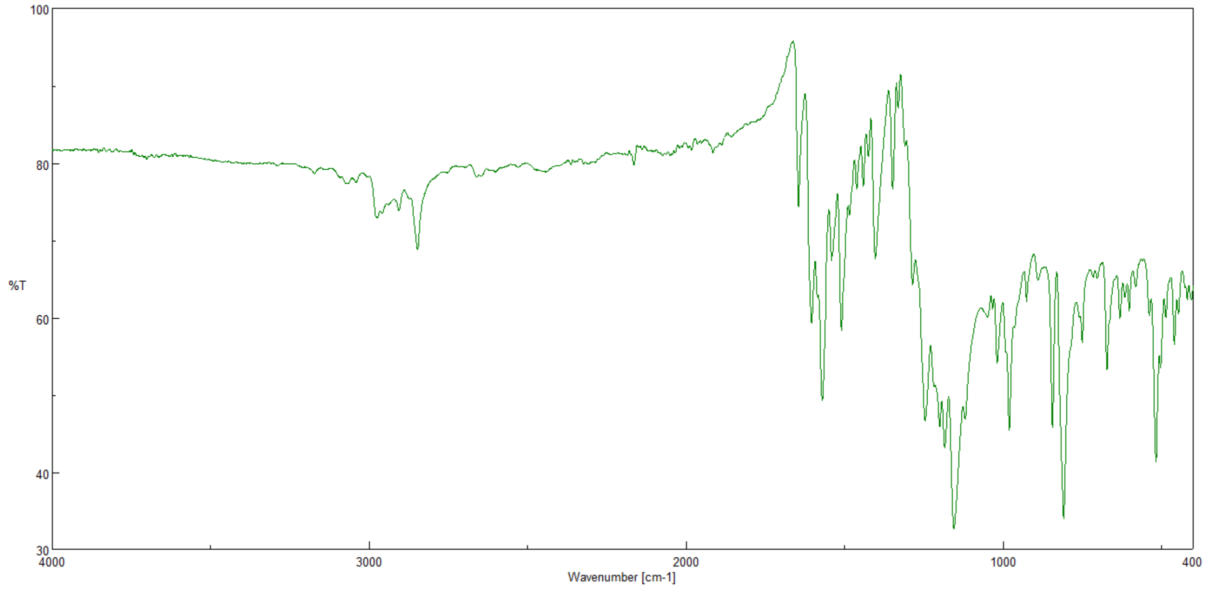

Figure S31. FTIR spectrum of compound **9**

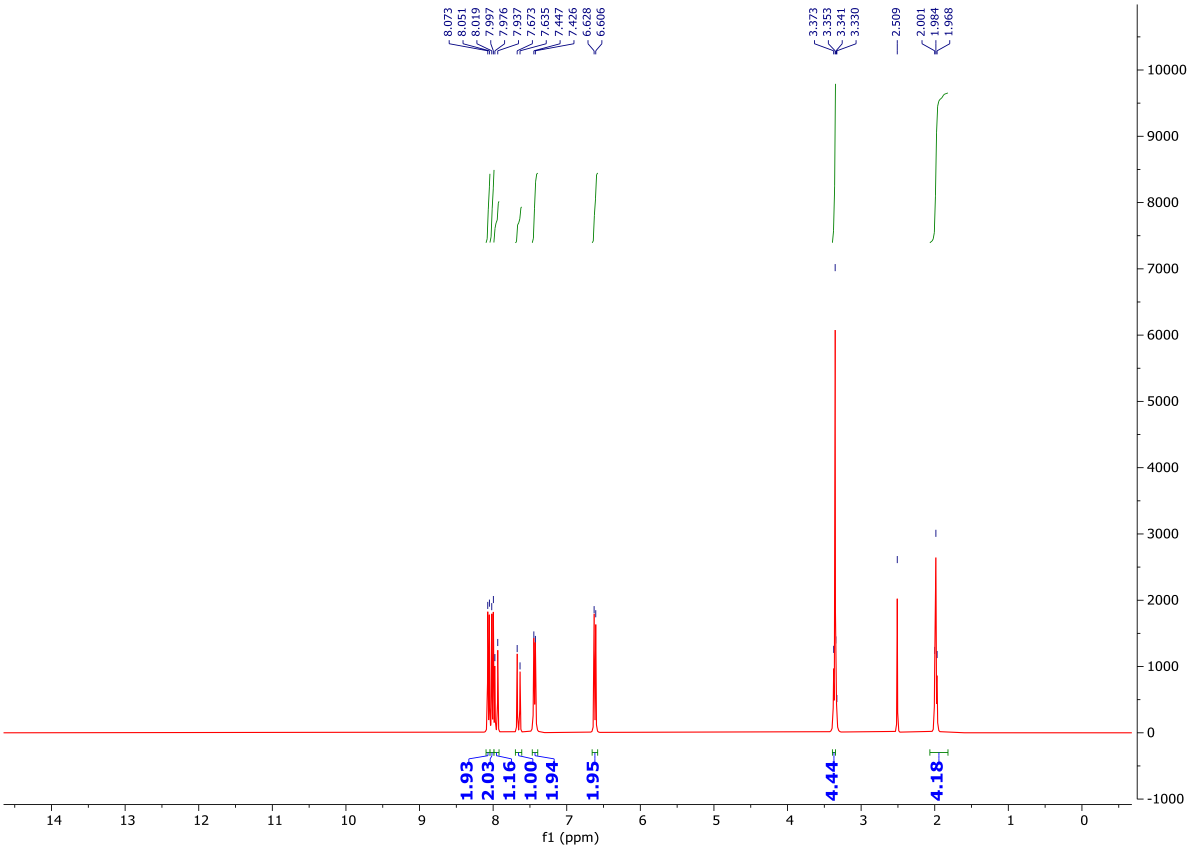

Figure S32. ^{1}H NMR spectrum of compound **9**

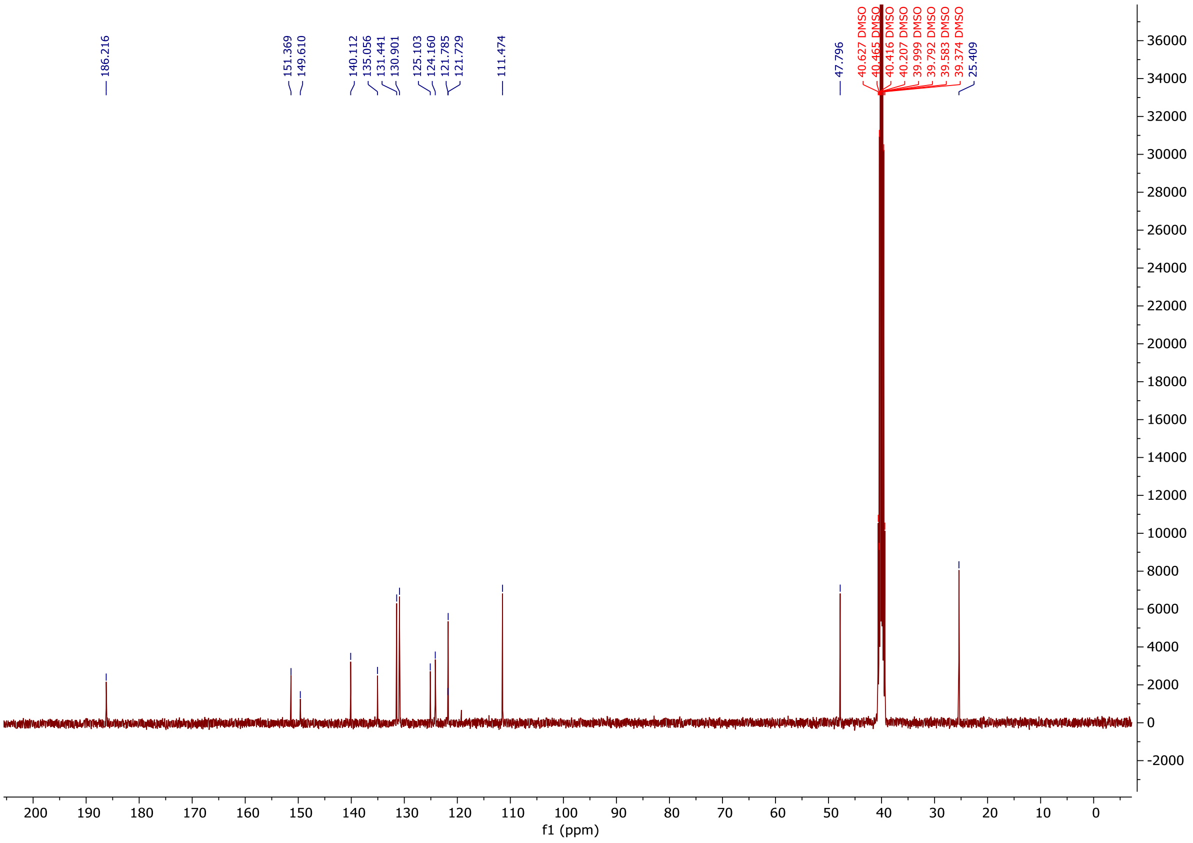

Figure S33. ^{13}C NMR spectrum of compound **9**

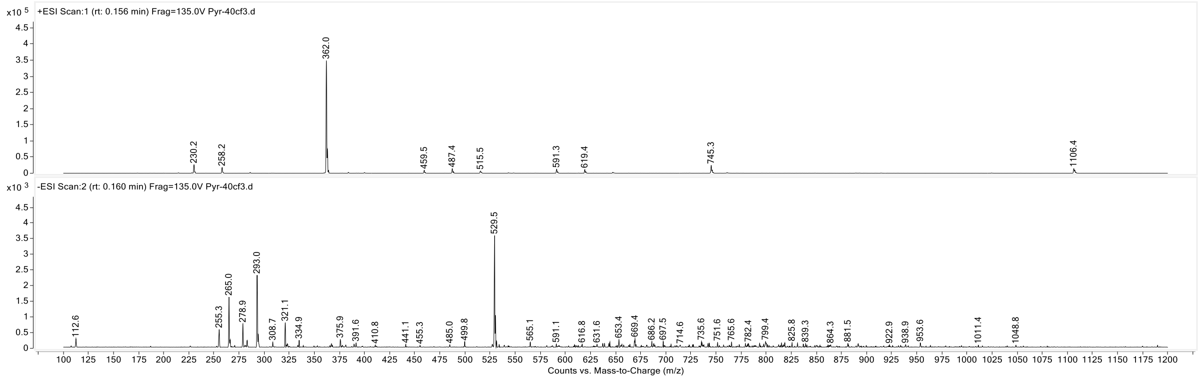

Figure S34. Mass spectrum of compound **9**

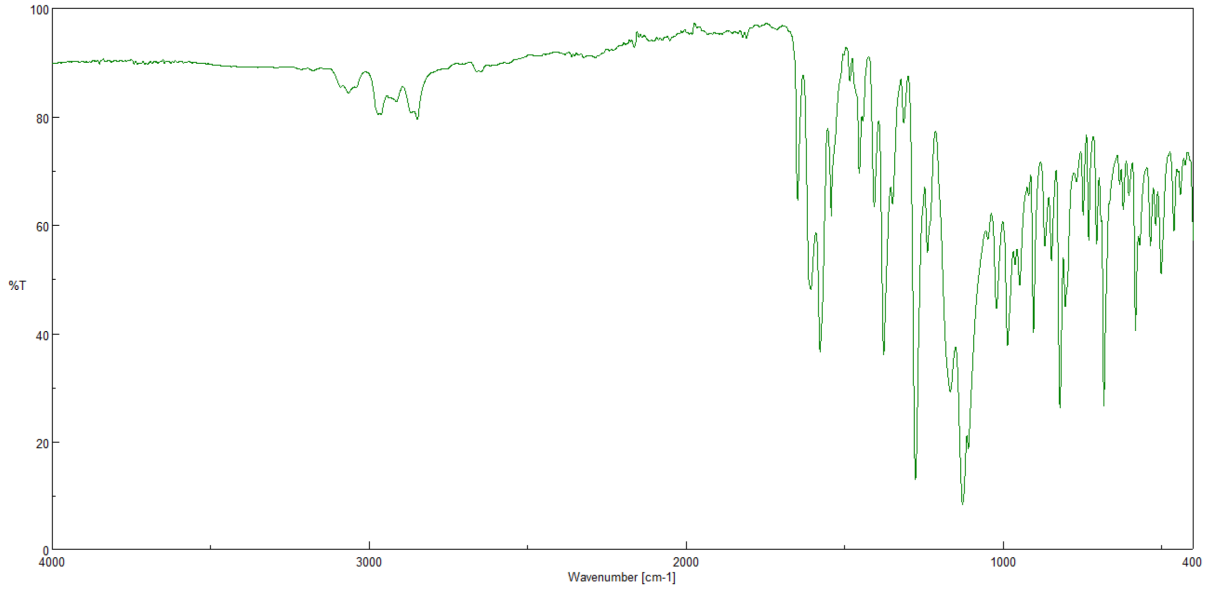

Figure S35. FTIR spectrum of compound **10**

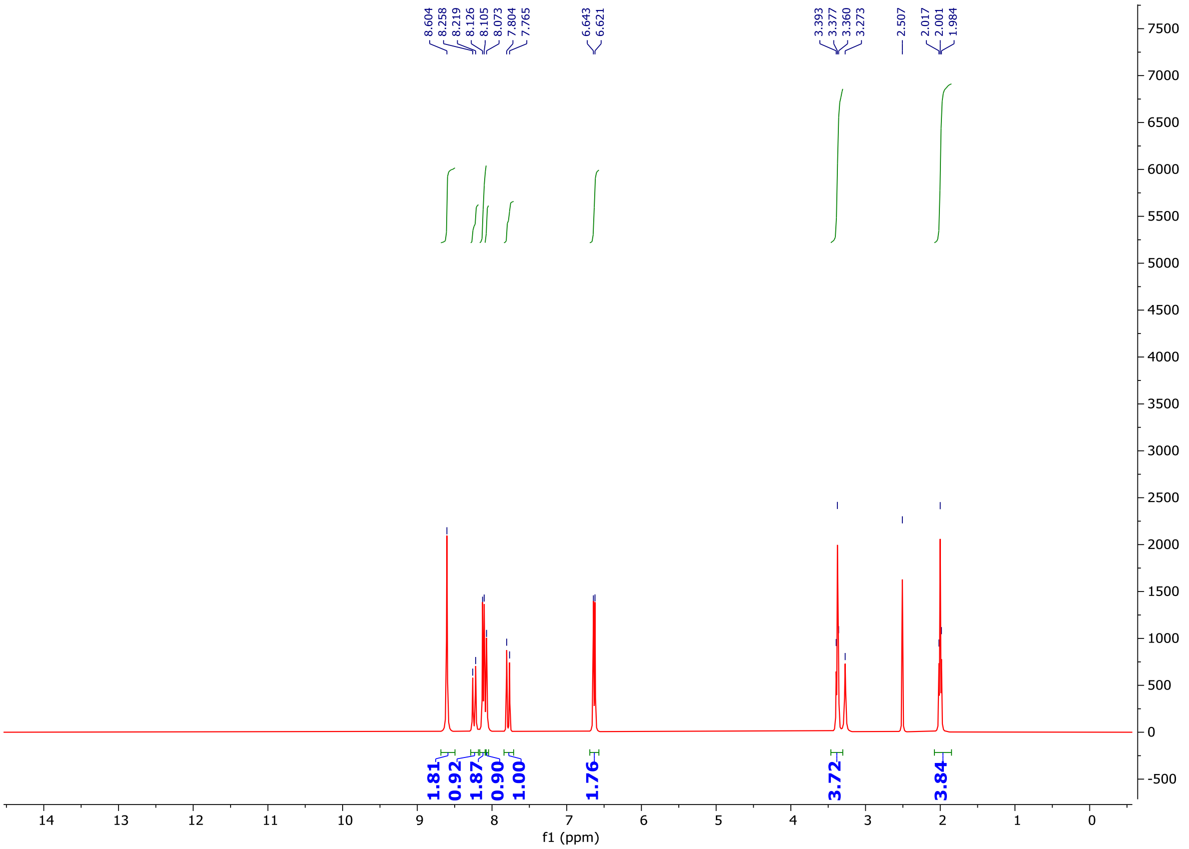

Figure S36. ^{1}H NMR spectrum of compound **10**

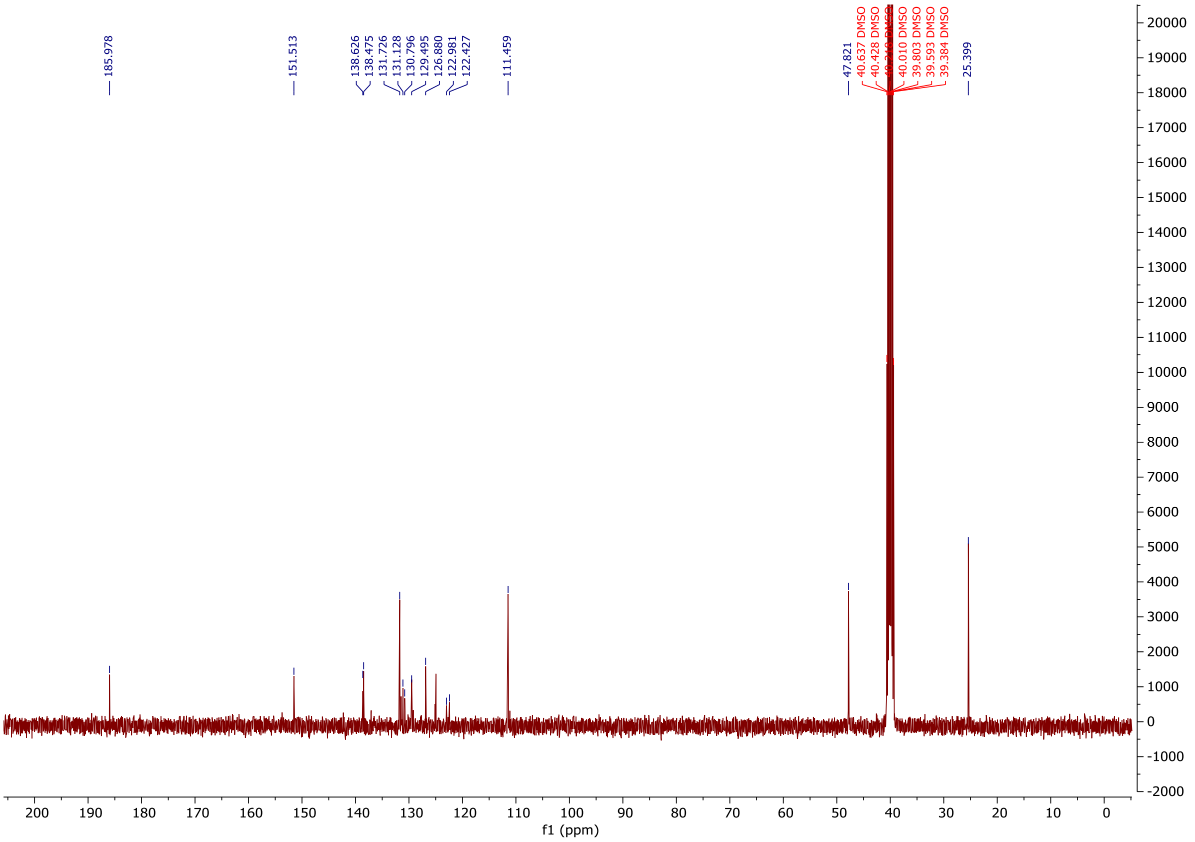

Figure S37. ^{13}C NMR spectrum of compound **10**

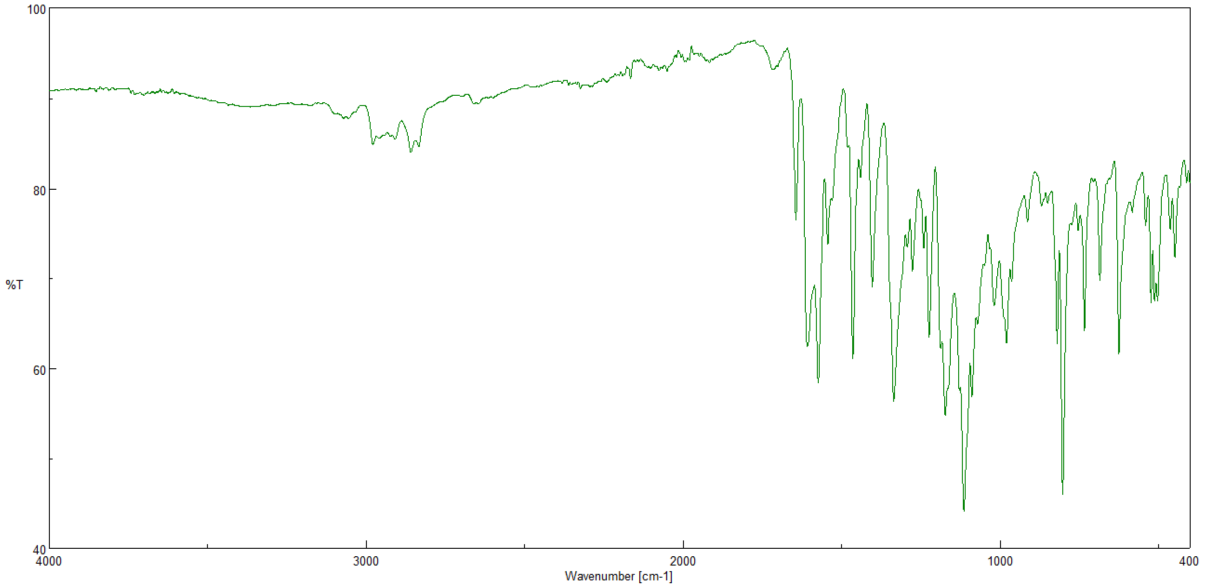

Figure S38. FTIR spectrum of compound **11**

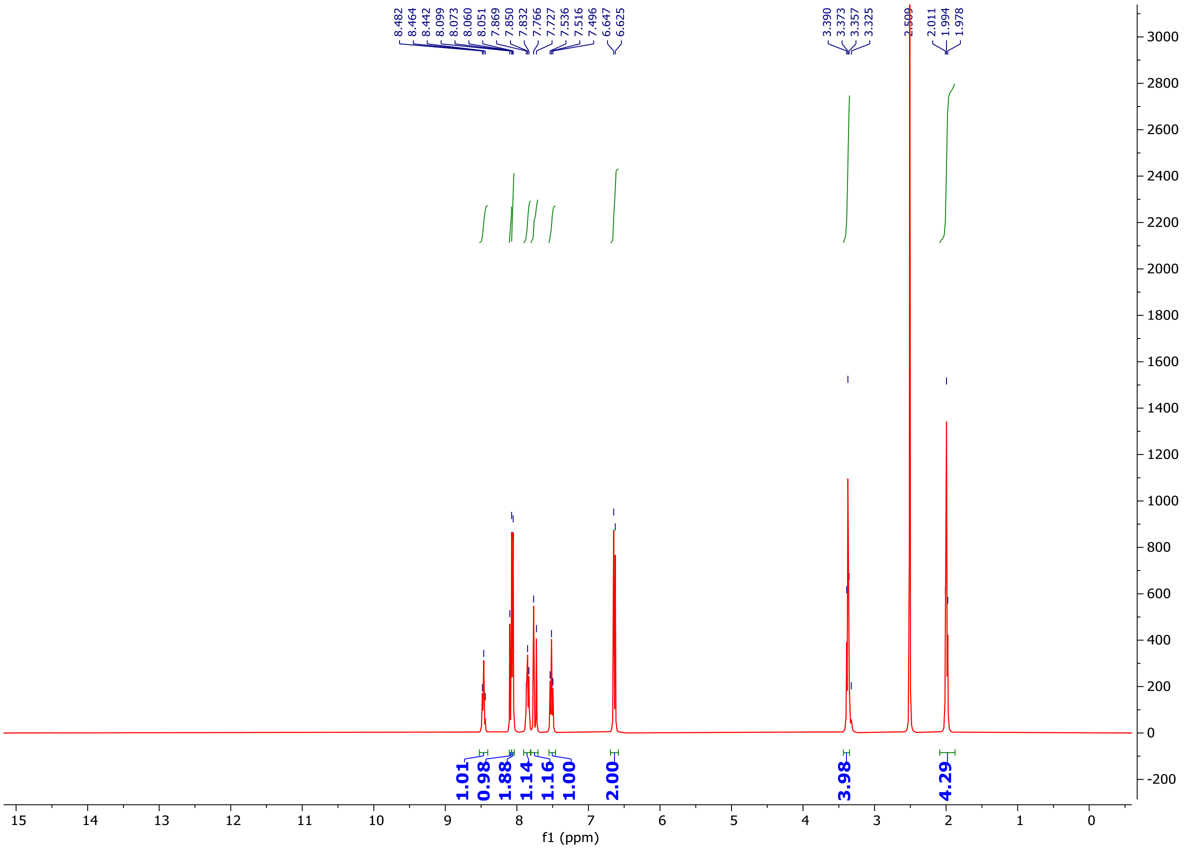

Figure S39. ^{1}H NMR spectrum of compound **11**

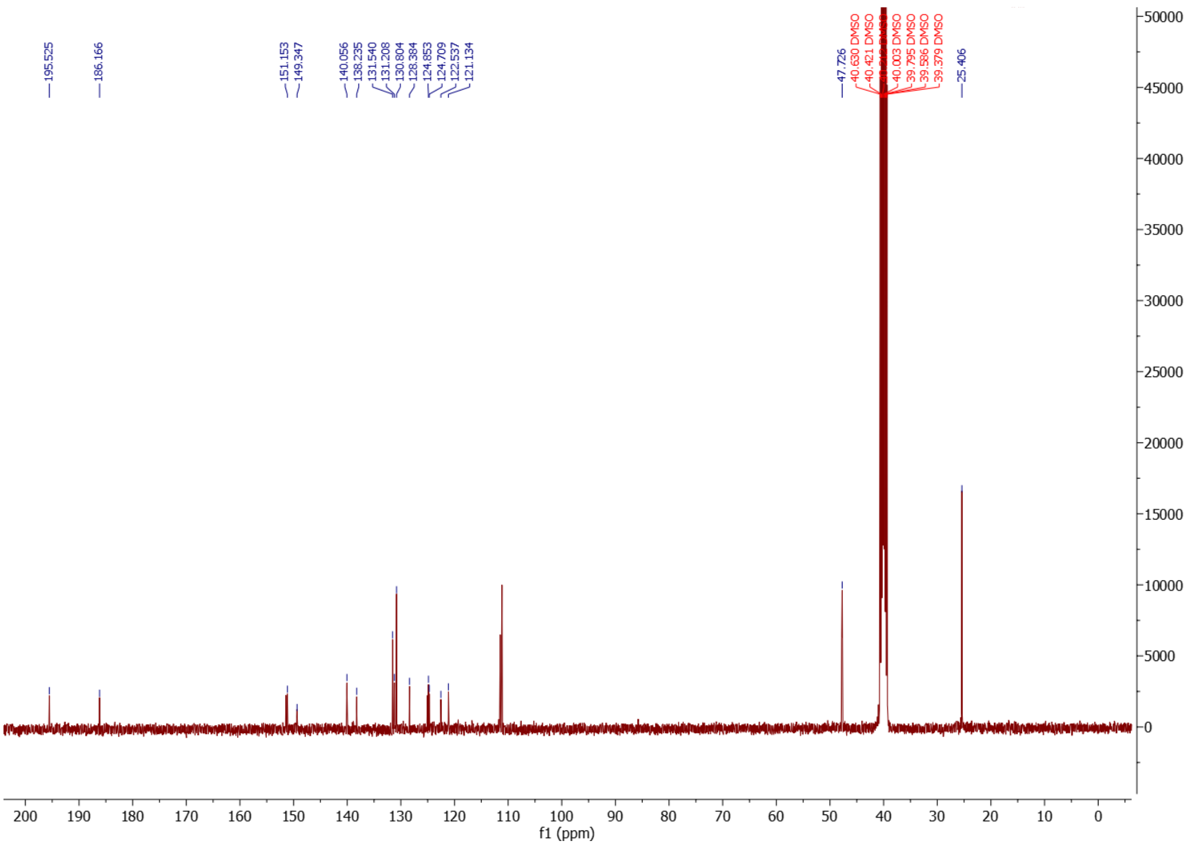

Figure S40. ^{13}C NMR spectrum of compound **11**

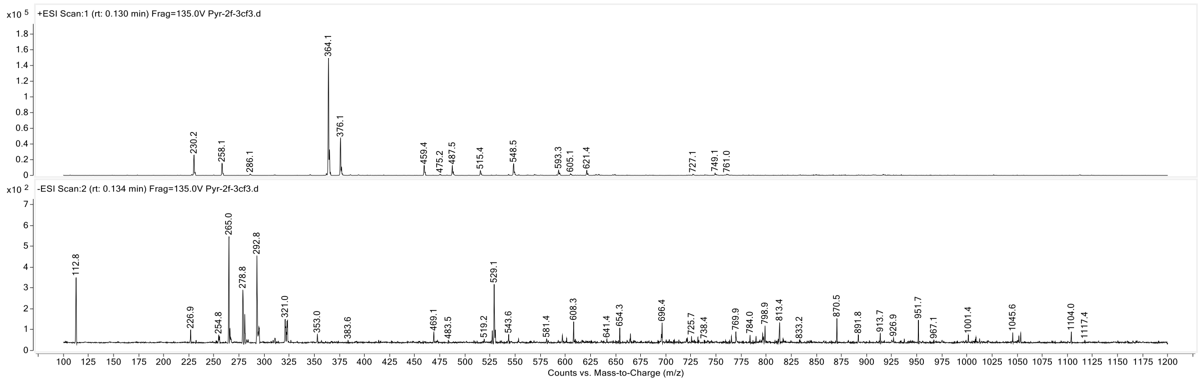

Figure S41. Mass spectrum of compound **11**

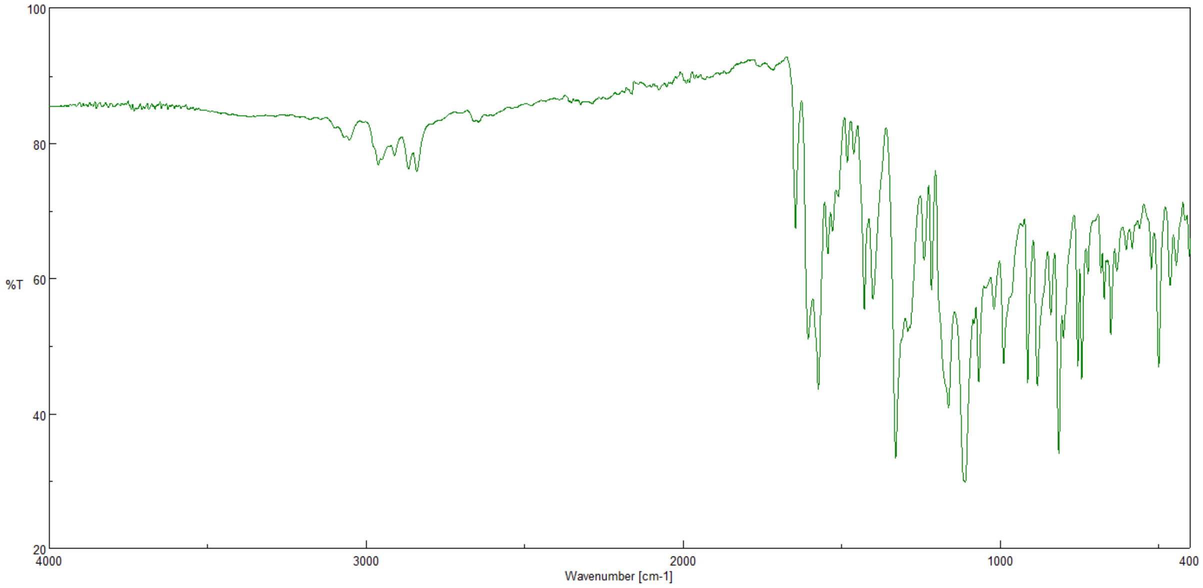

Figure S42. FTIR spectrum of compound **12**

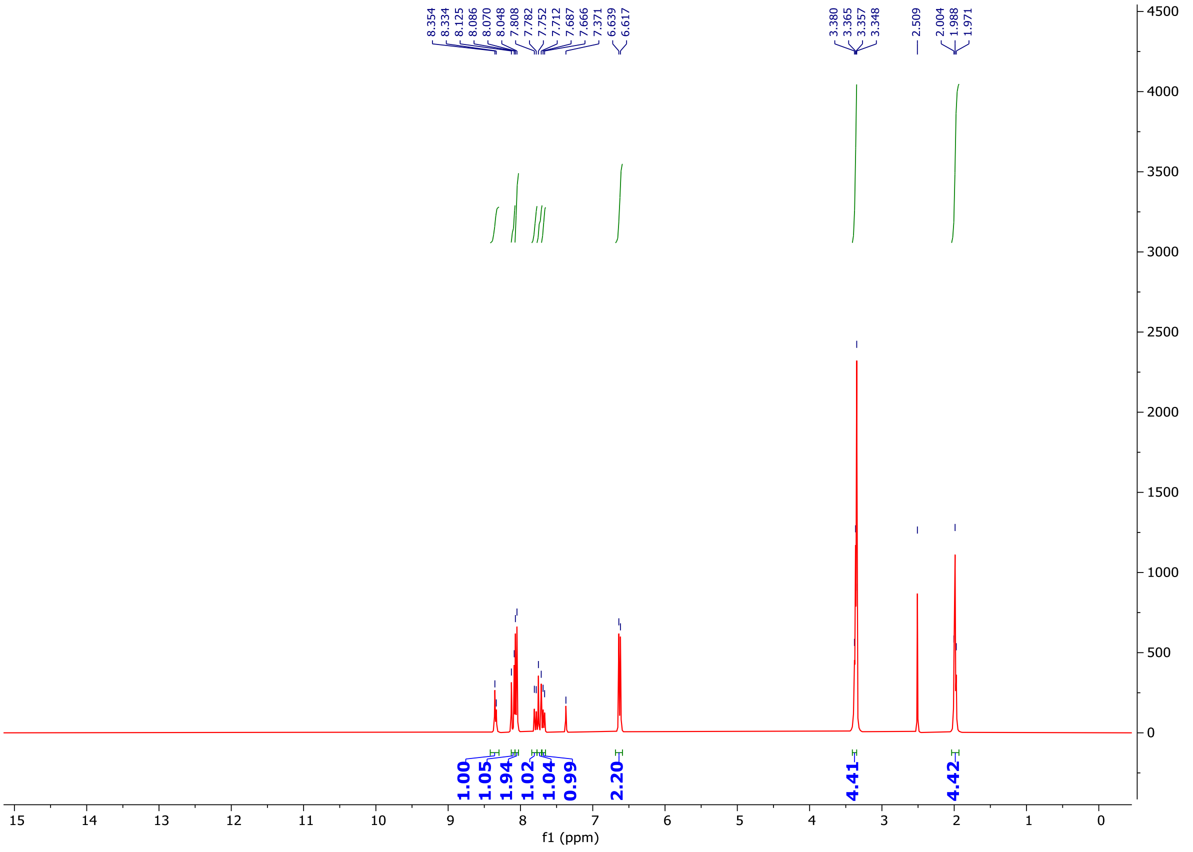

Figure S43. ^{1}H NMR spectrum of compound **12**

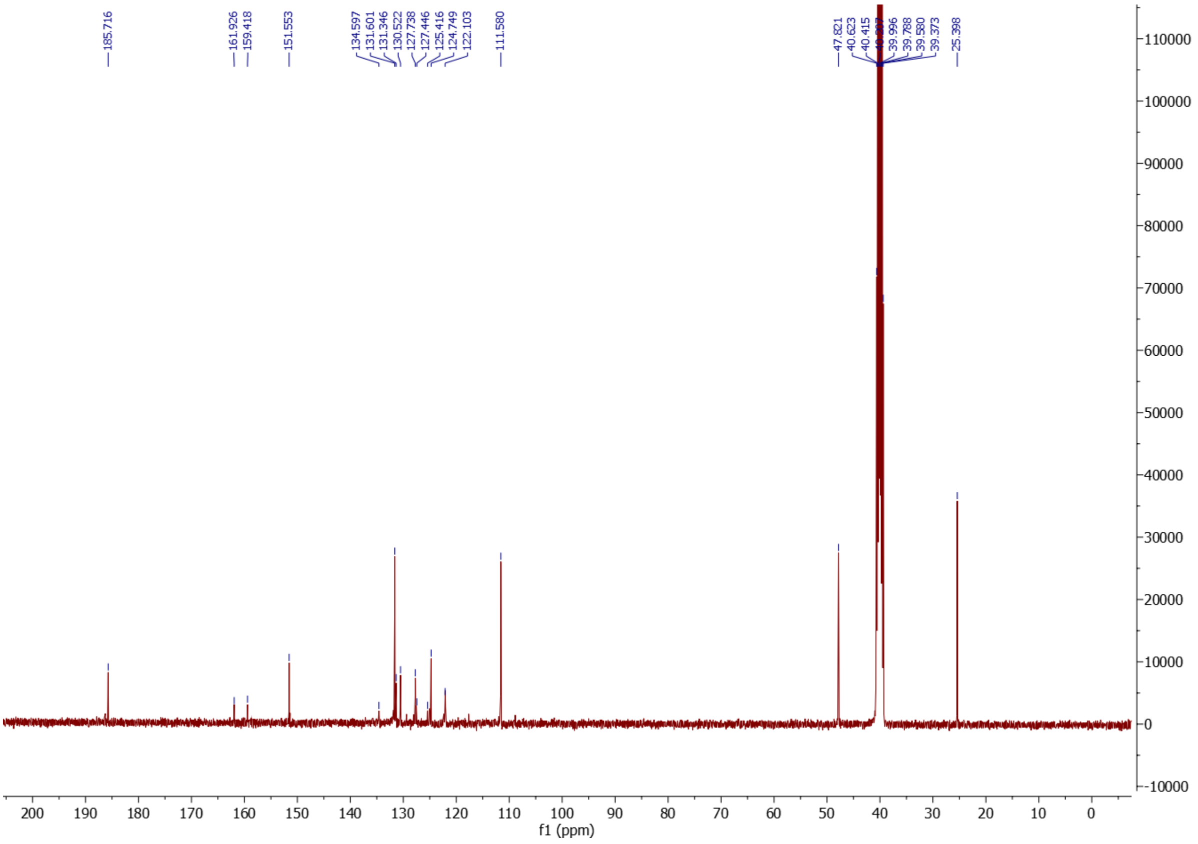

Figure S44. ^{13}C NMR spectrum of compound **12**

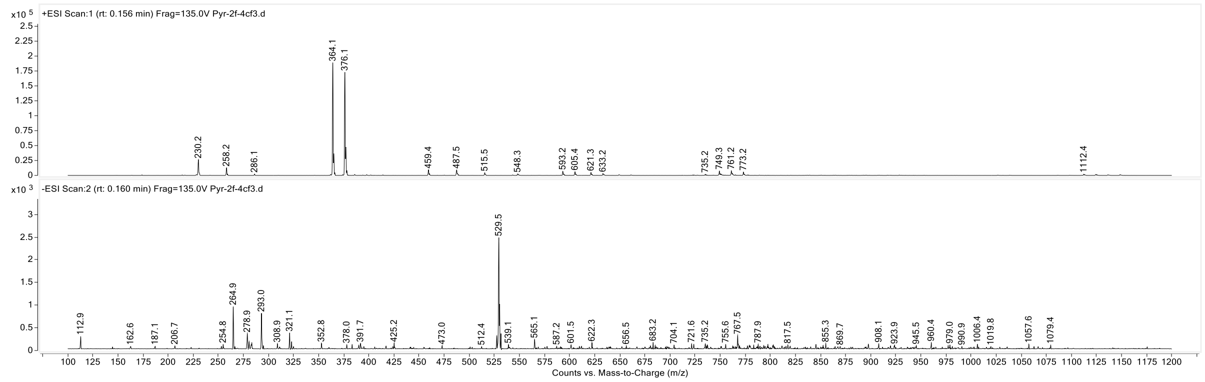

Figure S45. Mass spectrum of compound **12**

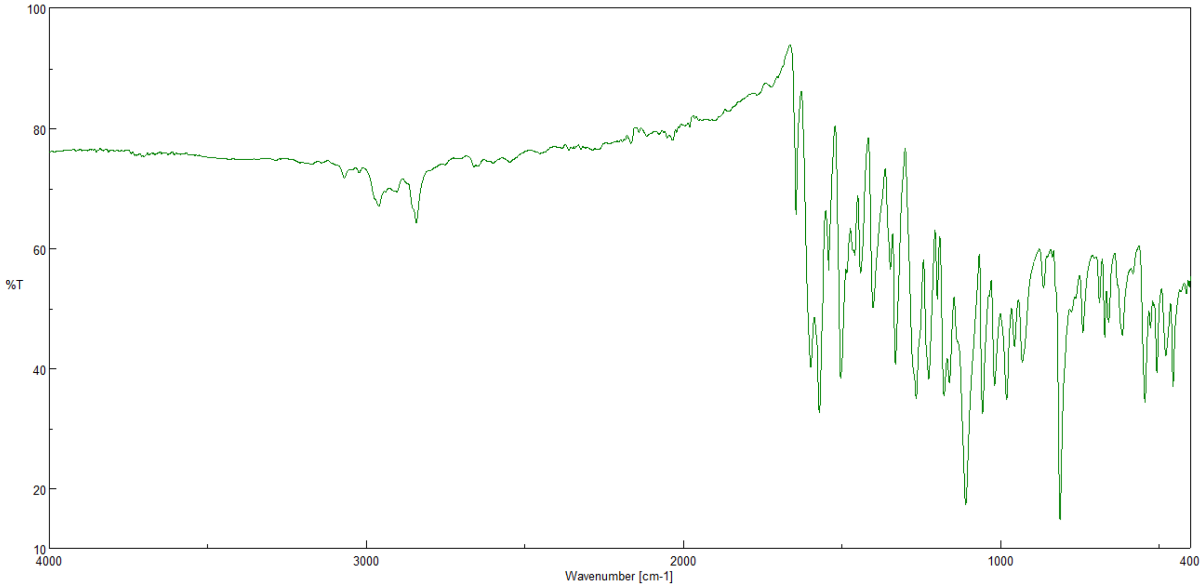

Figure S46. FTIR spectrum of compound **13**

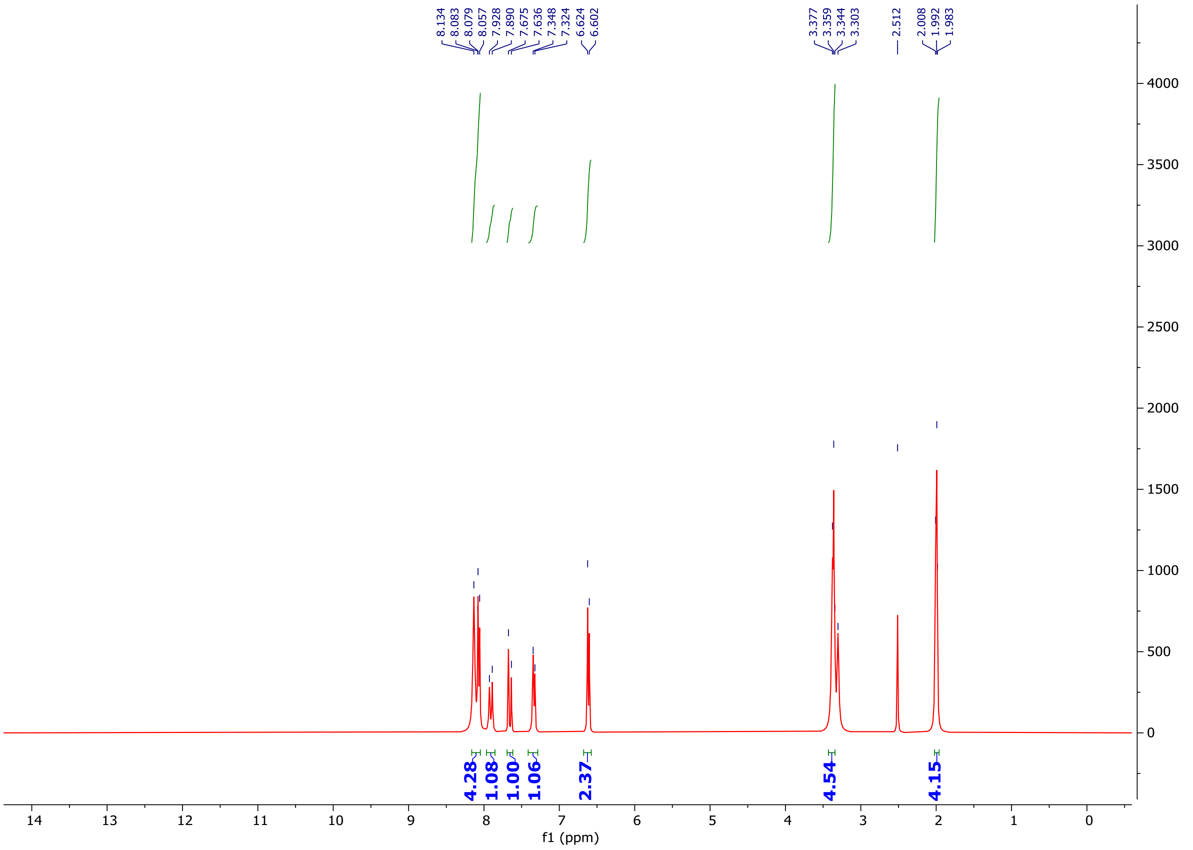

Figure S47. ^{1}H NMR spectrum of compound **13**

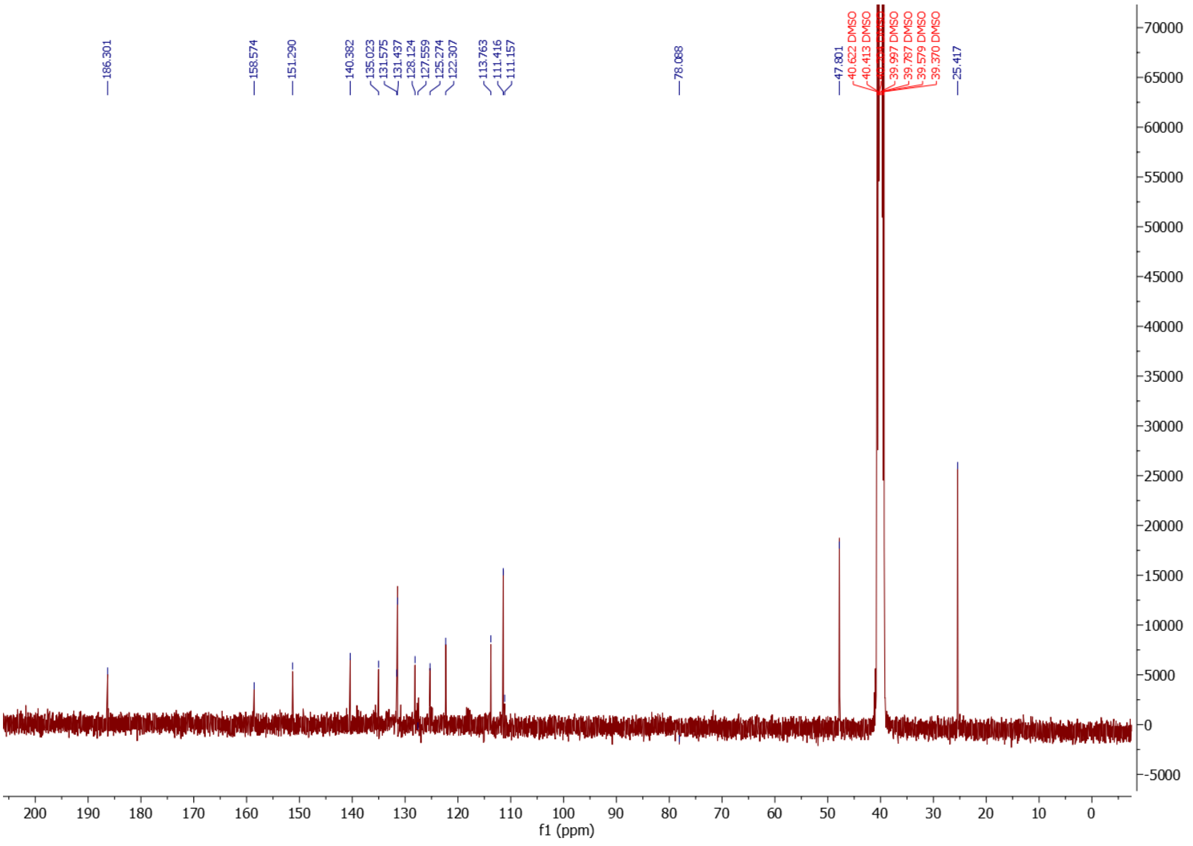

Figure S48. ^{13}C NMR spectrum of compound **13**

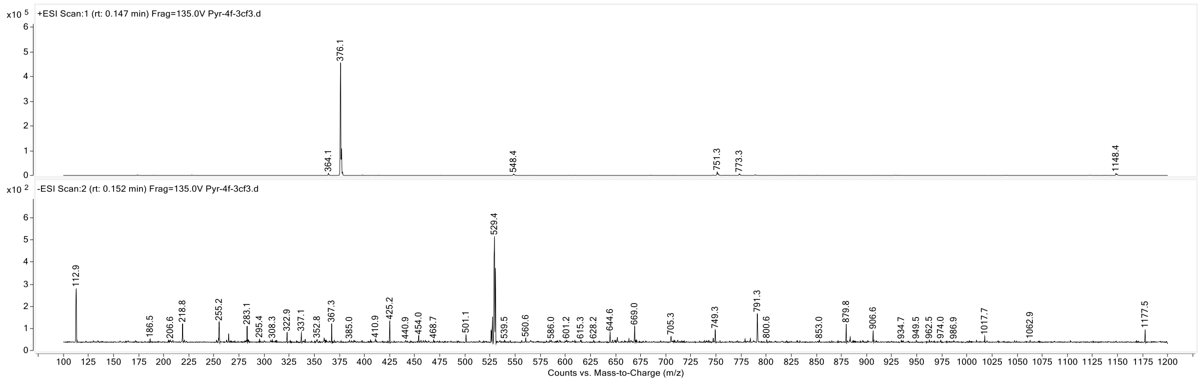

Figure S49. Mass spectrum of compound **13**
